# Generalised interrelations among mutation rates drive the genomic compliance of Chargaff's second parity rule

**DOI:** 10.1093/nar/gkad477

**Published:** 2023-06-09

**Authors:** Patrick Pflughaupt, Aleksandr B Sahakyan

**Affiliations:** MRC WIMM Centre for Computational Biology, MRC Weatherall Institute of Molecular Medicine, Radcliffe Department of Medicine, University of Oxford, Oxford, OX3 9DS, UK; MRC WIMM Centre for Computational Biology, MRC Weatherall Institute of Molecular Medicine, Radcliffe Department of Medicine, University of Oxford, Oxford, OX3 9DS, UK

## Abstract

Chargaff's second parity rule (PR-2), where the complementary base and k-mer contents are matching within the same strand of a double stranded DNA (dsDNA), is a phenomenon that invited many explanations. The strict compliance of nearly all nuclear dsDNA to PR-2 implies that the explanation should also be similarly adamant. In this work, we revisited the possibility of mutation rates driving PR-2 compliance. Starting from the assumption-free approach, we constructed kinetic equations for unconstrained simulations. The results were analysed for their PR-2 compliance by employing symbolic regression and machine learning techniques. We arrived to a generalised set of mutation rate interrelations in place in most species that allow for their full PR-2 compliance. Importantly, our constraints explain PR-2 in genomes out of the scope of the prior explanations based on the equilibration under mutation rates with simpler no-strand-bias constraints. We thus reinstate the role of mutation rates in PR-2 through its molecular core, now shown, under our formulation, to be tolerant to previously noted strand biases and incomplete compositional equilibration. We further investigate the time for any genome to reach PR-2, showing that it is generally earlier than the compositional equilibrium, and well within the age of life on Earth.

## Graphical Abstract

**Graphical Abstract:**
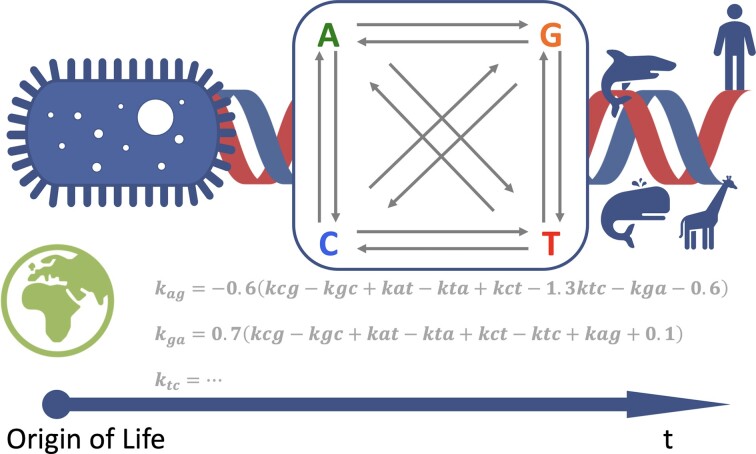

## INTRODUCTION

In 1950, Erwin Chargaff empirically observed that the four nucleotides are symmetrically abundant across the two strands in a double-stranded (ds) DNA molecule — the amount of adenine (A) is equal to the amount of thymine (T), and the amount of guanine (G) is equal to the amount of cytosine (C) — called the first parity rule (PR-1) (1). The explanation of this rule came with the 1953 discovery of the double helical structure of the DNA molecule with the G:C and A:T Watson–Crick base pairings at its core (2). In 1968, Chargaff separated the strands of the *Bacillus subtilis* genome into individual strands and discovered that the same sets of identities found for a double-stranded DNA (dsDNA) in PR-1 also hold true on each individual strand of the same molecule, i.e. the amounts of bases are equal in G/C and A/T pairs in each separate strand of a dsDNA as well, formulated as the second parity rule (PR-2) (3,4). This observation holds true almost universally across extant genomes and has even been extended to the frequency of higher-order oligonucleotides and their reverse complements, where the quantity of each k-mer is equal to the quantity of the reverse complement of that k-mer in the same strand (known as extended PR-2) (5–10). There are, however, exceptions to this rule for most organelles, single-stranded DNA (ssDNA) and RNA viruses, which do not comply with PR-2 (7,8,11–13). Being somewhat less clear from the structural and mechanistic considerations of DNA, PR-2 is very robust, hence requires an explanation that is as “crystal clear” and limiting as the Watson–Crick base pairing is for the PR-1. However, reviewing the scientific literature, an agreed consensus for the exact cause of Chargaff's second parity rule has not yet been determined. The current hypotheses behind PR-2 can be grouped into two schools of thought: intra-strand symmetry is (i) a feature of evolutionary convergence, and (ii) a feature of the primordial genome. To the best of our knowledge, the sub-categories of explanations are the following: (a) no strand biases for mutation and selection (6,14); selective pressure for the formation of stem-loop structures (15,16); statistics-based approaches (17–19); maximum entropy with the double helix constraints (9); strand inversion/inverted transposition/duplication of dsDNA followed by inversion (13,20,21); (b) original features of the primordial genome (as opposed to a feature of evolutionary convergence) (22).

Here, we start by re-examining the tolerance in genomic compliance to Chargaff's PR-2 in three kingdoms of species. Reviewing the major explanations proposed so far, we then adopt a completely assumption-free approach to find any possible link between mutation rate constants and PR-2. We demonstrate the contributory role of the equalities of mutation rates in dsDNA that are intrinsically present owing to its complementary double-stranded nature. Showing that, overall, these equalities hold true at a large scale, for at least the human genome, adding a weight on the no-strand-bias (NSB) assumption for PR-2, we also note a substantial variation allowed for the mutation rate constants around the paired equalities. Further examination coupled with symbolic regression and machine learning, leads us to a set of equations that define the universal and more permissive constraints on mutation rates. We demonstrate the better concordance of our equations with the experimental outcomes of genomes both compliant and non-compliant with NSB-driven equilibrium. Furthermore, we elaborate on the evolutionary convergence of genomes to PR-2, explaining why all dsDNA-based life complies with PR-2 at its present snapshot on Earth. The presented work reinstates the mutation rates as the major drivers behind the emergence of PR-2 even with strand biases in mutation rates and with an out-of-compositional-equilibrium state. This work demonstrates the simple principles behind the complex question and can serve as an important basis for future molecular evolution studies in genomics.

## MATERIALS AND METHODS

### General notes on the performed calculations

The developed workflows and analyses in this study employed the R programming language (https://www.r-project.org/). The resource-demanding computations were performed on a local Linux-based computing cluster at MRC WIMM, University of Oxford, by using nodes with 3 × 2.7 GHz 8-core E5-2680 Intel Xeon processors and 256 GB random access memory. The analytical derivations and checks have been done *via* the Mathematica software (https://www.wolfram.com/mathematica). The server application (GenomicPR2SimsWebApp) was written in R, using the Shiny library (https://cran.r-project.org/package=shiny) and a server application (https://shiny.rstudio.com). Figures were created with the R base, ggplot2 (https://ggplot2.tidyverse.org) and gridExtra (https://cran.r-project.org/package=gridExtra) libraries. Handling of the datasets was done by using the R base and tidyverse (https://cran.r-project.org/package=tidyverse) libraries.

### Processing of the eukaryotic, prokaryotic and DNA virus species

We have examined over 8000 genome sequences ranging from eukaryotes, prokaryotes, and DNA viruses. We accessed the Ensembl Genomes database *via* FTP (ftp://ftp.ensemblgenomes.org) and downloaded all genome sequences from, at the time of performing this work, the latest release for bacteria (ftp://ftp.ensemblgenomes.org/pub/bacteria/release-48) and eukaryotes (ftp://ftp.ensembl.org/pub/release-102/fasta/). For DNA viruses, we accessed the NCBI database (https://www.ncbi.nlm.nih.gov/labs/virus/vssi/#/find-data/virus) and downloaded all species categorised as “DNA viruses”. For the bacteria and eukaryotes, we downloaded the genome sequences in an iterative process *via* the R code. We used the Biostrings (https://bioconductor.org/packages/Biostrings/) library to calculate the nucleotide composition for all three kingdoms (Supplementary Figure S1). We note that a significant number of sequences had to be removed because of, for instance, duplicates of a given strain of a given species. Taking prokaryotes as an example, there were approximately 44,000 species in the original file from the Ensembl database, but we noticed that a single species could have thousands of entries in the file which would skew the species-wide analysis and yield inaccurate results. The same problem also occurred with eukaryotes and DNA viruses. Thus, the goal was to filter the files with the following methodology: in a group of species with the same name, we only saved the first occurring row of the given species name and repeat this process for the remainder. At the end of this process, we discarded many duplicates and filtered the data as consistently as we could, including manual filtering processes. Full details are on the corresponding GitHub repository.

### Processing of the human and chimpanzee genomes

Analyses involving the human genome were performed using the unmasked version of the human reference sequence hg19/GRCh37, as accessed from Ensembl (23) genome database (www.ensembl.org). We used the unmasked chimpanzee genome, version 2.1.4.75, for calculating the genomic base and dyad contents (Supplementary Note S1). The fasta files were downloaded from the Ensembl database. While also trying the masked genome, we noted only negligible differences on both single base and dyad contents. For counting dyads, both segmentation (into dimers) and sliding window methods were used, again with no significant differences noted.

### Mutation rate constants for use in simulations

The mutation rate constants used in this work were all brought from previous work using the Trek methodology (24), where the human genome mutation rates were revealed through the remnants of LINE-1 elements in a single genome manner. We also re-calculated such rate constants using LINE-1 elements that reside in only +, and in only -, strands to arrive at strand-specific but still genome-wide overall values for mutation rates. Strand-symmetry accounted for mutation frequencies for the following species: *Homo sapiens*, *Escherichia coli*, *Caenorhabditis elegans*, *Drosophila melanogaster, Aotus thaliana* and *Saccharomyces cerevisiae* were obtained from (25). All such frequencies were next brought into a scale of our rate constants for use in our simulation models. In the supplementary materials of (24), Supplementary Figure S3 shows the relationship of the mutation rate constants for the human genome obtained with the Trek methodology versus the mutation frequencies reported from other datasets. We replicated Supplementary Figure S3a (24) with the obtained frequencies from other species and the mutation rate constants from the Trek methodology and performed a linear regression through the zero origin. This resulted in the $k\ = \ 2.831f$ equation. As we were interested in the genomic average mutation rate constants in a time domain, we used an assumption that this quantitative relationship between the mutation rate constants and mutation frequencies holds for the other (non-human) five species. Thus, the frequency values, obtained from the literature, were used as inputs (*f*) in the above equation to generate the average mutation rate constants (*k*) in the time domain for our simulation.

### Generation of simulations

The numerical analyses were performed using the R programming language with its deSolve library (https://cran.r-project.org/package=deSolve) for solving the system of differential equations. Random sampling of the mutation rate constants for the continuous uniform distribution was done with the runif() function, and the truncated normal distribution was done with the rtruncnorm() function from the truncnorm library (https://cran.r-project.org/package=truncnorm). All simulations were run in parallel execution using the foreach() (https://cran.r-project.org/package=foreach) and doParallel() (https://cran.r-project.org/package=doParallel) functions. For reproducibility, we set the initial seed to 1 and used the doRNG (https://cran.r-project.org/package=doRNG) library for the parallel child processes in our foreach loops.

### Defining the equilibration time periods

Simulation of the replicates was done using symmetry-constrained mutation rate constants drawn from a truncated normal distribution using the rtruncnorm() function from the truncnorm library in R. Where the equilibrium was needed, we carried out the following five-step process to define a genome equilibration tolerance for the simulation: (A) calculate the sum of the differences of base contents divided by four; (B) repeat the simulation 100,000 times; (C) calculate the mean fluctuation of (A) for the 100,000 times; (D) check that the mean fluctuation is not greater than 1% of 25 of the mean fluctuations that we define as the EQtolerance; (E) calculate the absolute difference of each base content and find the difference between the maximum and minimum. When this time is less than or equal to the EQtolerance, the genome was considered equilibrated. Independently from the equilibration compliance, PR-2 compliance was defined as the time when both the *C*_*GC*_ versus*C*_*AT*_ skew are below their respective tolerance values obtained for each of the three kingdoms: eukaryotes, prokaryotes, and DNA viruses, as discussed below.

## RESULTS AND DISCUSSION

### The universality of PR-2 in dsDNA genomes of different species

**Figure 1.**
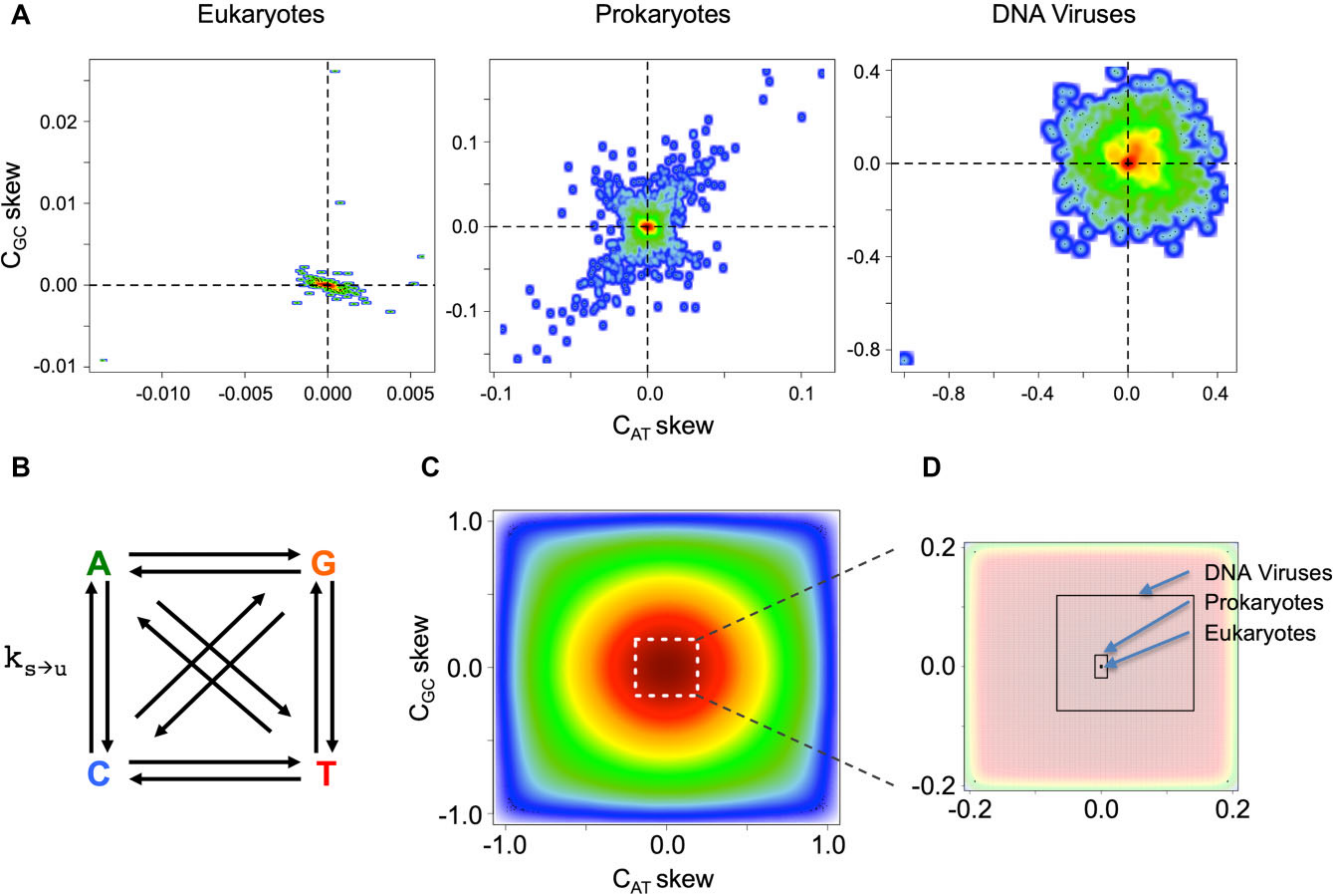
Chargaff's PR-2 in different species and the unconstrained mutational network model. (**A**) The plots for the base content *C*_*GC*_ versus *C*_*AT*_ skews for each of the three kingdoms analysed in this work (see Supplementary Figure S2 for the exact mean and standard deviation values). The $\bar{x} \pm s$ range for each kingdom was used for the naturally observed PR-2 fluctuation while analysing the results of the simulations. (**B**) The most general mutational network model, where all the 12 ${k}_{s \to u}$ rate constants are unique and independent from each other. The system was numerically solved to produce the time evolution of genomic base content within a 4.28 byr period, the current maximum estimate of the age of life on Earth (36). (**C**) The 2D kernel density estimate scatterplot, showing the distribution of *C_GC_* and *C_AT_* skews from the outcome of the simulation (colours vary with decreasing occurrence frequency from red to blue). There, the white dotted box indicates the area zoomed in (D). (**D**) Zooming to demonstrate the strict PR-2 compliant zones for each of the three kingdoms, where dimensions of the boxes represent the total $\bar{x} \pm s$ range for the corresponding axis and kingdom (see section “The universality of PR-2 in dsDNA genomes of different species" in Results and Discussion for exact values).

The re-examination of the universal nature of the Chargaff's PR-2 compliance of dsDNA on an up-to-date collection of genomes is shown in Figure 1A, where we bring the plots for *C_GC_* versus *C_AT_* skews, as defined by (G – C)/(G + C) and (A – T)/(A + T), for the genomes of species across the eukaryotes, prokaryotes, and DNA viruses. Strong adherence to PR-2 can be noted in all kingdoms (Figure 1A) with the intra-strand symmetry phenomenon holding true across the extant dsDNA genomes (standard deviations: 1.908 × 10^−3^, 1.907 × 10^−2^, 9.666 × 10^−2^ for *C*_*GC*_, and 1.178 × 10^−3^, 9.400 × 10^−3^, 1.041 × 10^−1^ for *C_AT_* across eukaryotes, prokaryotes and DNA viruses, respectively), except for the organelle and other ssDNA genomes (7–10). While focusing on the low-density scatter in the *C*_*GC*_ and *C*_*AT*_ skews, the viral DNA genomes are narrowly spread, while the eukaryotic organisms form a dense and localised cluster. In prokaryotes, the low-density scatter in the plots is not only more diffuse, as compared to eukaryotes, but there seems to be an emerging diagonal pattern, linked, based upon our experimentations, with the G+C content of a genome.

Based on these plots, we can infer the allowed PR-2 fluctuations for each of the three kingdoms. This allows the definition of “tolerance” values for PR-2 compliance, essential for our further studies. We examined the tolerance values from the *C_GC_* and *C_AT_* skews as opposed to the *C_G/C_* and *C*_*A/T*_ ratios (see Supplementary Figure S2), because, in the former case, the range of allowed values is limited between $[ {0,\ 1} ]$ while the mathematical nature of the *C*_*G/C*_ and *C*_*A/T*_ ratios allow a biased range from $[ {0,\infty } ]$. The preferential regions of PR-2 compliance were defined from the analysed experimental genomes for each kingdom, by taking the mean value of the skews (usually tending to 0) and one standard deviation allowed at both sides of the mean. For *C_GC_* skews, the PR2-compliant preferential fluctuation regions were 7.780 × 10^−5^ ± 1.908 × 10^−3^, 1.425 × 10^−4^ ± 1.907 × 10^−2^ and 2.262 × 10^−2^ ± 9.666 × 10^−2^ for eukaryotes, prokaryotes and DNA viruses respectively. For *C_AT_* skews, the ranges were 1.096 × 10^−5^ ± 1.178 × 10^−3^, –1.386 × 10^−5^ ± 9.401 × 10^−3^ and 3.668 × 10^−2^ ± 1.041 × 10^−1^ in the same order as above.

### An overview of the past theories to explain PR-2

One of the earliest attempts to explain the emergence of PR-2, and one that was initially well received, was the hypothesis of no-strand-bias (NSB) conditions (6,14). In 1995, Sueoka determined that, when there is no bias in mutation and selection between the complementary strands, base mutation may explain the parity phenomenon (14). For instance, for a $C \to T$ mutation in one strand of a dsDNA under NSB, the rate for the $C \to T$ mutation on the other, complementary, strand can be considered the same. Due to the first parity rule, $C \to T$ mutation leads to a $G \to A$ mutation on the complementary strand. Therefore, the $C \to T$ and $G \to A$ mutation rates match on the same strand too, since $G \to A$ mutation is the same $C \to T$ mutation on the complementary strand, and the rates of $C \to T$ mutations on both strands match under NSB. As a result, a mutation network of 12 unique mutation rates can be simplified down to only six independent rates (14). In the same year, Lobry derived a set of differential equations that showed that, at equilibrium, the base frequencies within each strand are equal, i.e. resulting in PR-2 (6). While a mutation network with NSB was a working hypothesis by both Sueoka and Lobry, it was shortly afterwards rejected by Lobry because the equations assume that both the mutation rates and the resultant genomes are constantly at equilibrium. It suggests that any deviation from PR-2 could either mean that the model is wrong or that the system is out of equilibrium. Considering known examples of genomes being out of equilibrium (26–28), as well as mutational processes bearing some strand asymmetries (29), this hypothesis cannot be the universal explanation for PR-2 (27,28). While Sueoka and Lobry used a base-mutation model to explain the cause of PR-2, in the same year, Forsdyke proposed that PR-2 is a product of selective pressure favouring point mutations that assist in the formation of stem-loops (15,16). He argued that such formations may be advantageous for recombination events, thus playing an important role in driving the symmetry between complementary oligonucleotides. However, similar to Sueoka and Lobry, Forsdyke's hypothesis has also been challenged. Zhang *et al.* suggested that most oligonucleotides (with sequence lengths of five and longer) do not have a reverse complement nearby in the genome sequence and thus the short-range contribution of local stem-loop potential by complementary oligonucleotides is limited (30). Chen *et al.* also challenged the stem-loop hypothesis for human chromosomes (31).

Albrecht-Buehler proposed that PR-2 is the inevitable, asymptotic product of the cumulative action of proposedly ever-repeated inversions and inverted transpositions in genomes (20,21). He extended his inversion/transposition hypothesis by observing that these mechanisms also generated a universal triplet profile for most of the evolving organisms, i.e. the frequency of triplet oligonucleotides is almost equal to the frequency of their reverse-complement, in compliance with the extended PR-2. He further explained that the evolutionary advantage of an almost universal genome may be to help with horizontal gene transfer between species that are vastly different from one another. However, the biological plausibility of such ever-repeated inversions in the present limited evolutionary timescale is questionable, and it is known that the point mutations occur at an order of magnitude more frequently than canonical indels (32,33). Thus, if PR-2 can be explained based on point mutations, that explanation should have precedence over the explanation based on a specific subtype of indels. His conclusions from this paper have also been challenged by Zhang *et al.* who found that at least among prokaryotic genomes, two common triplet profiles existed, one for low-G+C and the other for high-G+C content genomes (34). Similar to Albrecht-Buehler's hypothesis, Okamura *et al.* proposed that inversions, which may or may not be preceded by duplications, may be a major contributor to the phenomenon of the intra-strand parity rule (13). They found that when subjecting the human mitochondrial DNA to inversions *in silico*, the frequencies of specific trinucleotides and their reverse complementary oligonucleotides became broadly symmetrical, suggesting that this mechanism could lead a sequence to parity. It is worthy to note, however, that the biological and mechanistic plausibility of such repeated inversions and transpositions as a widespread phenomenon in genomes remains to be confirmed.

Several scientists have used statistics-based approaches to explain the cause of PR-2. For instance, Baisnée *et al.* (7) demonstrated that using simple, first-order Markov models to model biological sequences was not enough to explain symmetries in dsDNA genomes. Instead, the emergence of symmetry may be explained through a combination of mechanisms, such as inversions, among other possibilities. Hart *et al.* (17) showed that the Gibbs distribution, which is associated with the reverse complementary relationship between the nucleotide interactions on each strand of dsDNA, can explain PR-2. An observation made by Shporer *et al.* (8) showed that when we consider a genome of a given length *L*, then the highest k-mer (substrings of length *k*) for which PR-2 holds true is given by 0.7ln(*L*), thereby demonstrating that the extended PR-2 rule holds true up to a certain length of a k-mer. However, their observation has been generalised in a recent paper by Fariselli *et al.* (9), which does not impose an upper limit to the k-mer length for the PR-2 to hold true. They proposed a solution using the maximum entropy with the double helix constraints, which predicts that at equilibrium, in a long-enough dsDNA sequence, the probability of occurrence of a k-mer and its reverse complement tend to be the same. In other words, the leading force shaping the symmetrical DNA duplex structure is randomness, rather than biological/environmental pressures. A recent paper by Cristadoro *et al.* (10) showed that Chargaff's parity rules are not the only symmetry phenomena present in the genetic sequences of *Homo sapiens*; instead, there exists a hierarchy of symmetries at different structural scales. These observations are similar across all nuclear chromosomes of *Homo sapiens*, thus suggesting that some mechanisms that shape both the structure and symmetry work at the same time in the chromosomes, the leading mechanism of which is currently unknown. They further investigated the evolution of the genome dynamics, in which they developed a model to mimic the action of inversion/transpositions of transposable elements on DNA, originally motivated by Albrecht-Buehler's simulations (20,21). The results show that the simultaneous occurrence of symmetry and structure is an emergent property of the dynamics of transposable elements in DNA sequences (9). In particular, they found that symmetry and structure change differently depending on the time scales, i.e., for a large time interval, they were able to reproduce the same structure and symmetry as in extant genome sequences.

An alternative school of thought on the cause of PR-2 was proposed by Zhang *et al.* (22). They suggest that the origin of strand symmetry and oligonucleotide frequency conservation has existed from the very beginning of the genome evolution and thus, compositional features of the genome would be “relics” of the primordial genome as opposed to a feature of evolutionary convergence — the umbrella hypothesis of the previously discussed scientific literature. Zhang *et al.* argued that the primordial genome would be composed of approximately equal amounts of uniformly distributed forward and their reverse-repeated sequences, thus resulting in a strand-symmetric genome where frequency conservation is a consequence of it, although they note that the degree of strand symmetry decreases with increasing order of oligonucleotides.

### Assumption-free approach to look for a link between mutation rates and PR-2

Of all the prior hypotheses reviewed above, we can see that the NSB approach (6,14) was the one that was proposing a strong constraint at the core of the molecular processes that govern dsDNA composition, thus being as strict as the observation of the PR-2 compliance in genomes is. However, the approach was dismissed (27,28) due to the expected aberrations from NSB (29,35) and/or compositional equilibrium (26) regionally, in shorter dsDNA spans, and in some species. In this work, we thus revisit the link between the mutation rate constants and PR-2, but with no initial assumption on the mutation rates and their equalities whatsoever. We numerically calculated the base content coming out of a 4.28 billion years (byr, the estimated age of life on Earth (36)) dynamics of the unconstrained cross-mutational network, where every base mutation is assumed to have an independent rate constant, i.e. each conversion arrow in Figure 1B to have an unconstrained $k_{s\to u}$ value, where the subscript denotes the mutation of the base $s$ into $u$.

We simulated 25 million systems starting from a completely random genome (0.25 for $\{ {A,\ T,\ G,\ C} \}$ base compositions) to obtain enough PR-2 compliant samples based on our PR-2 tolerance ranges calculated in section “The universality of PR-2 in dsDNA genomes of different species” of Results and Discussion for the eukaryotes, prokaryotes, and DNA viruses. We also simulated systems A) starting from random $\{ {A,T,G,C} \}$ contents within a range of allowed base content values in eukaryotic and prokaryotic organisms for the first two randomly sampled bases, with the remaining two bases being sampled from $1 - remainder$ for the four bases to always sum to 1 for a complete genome, and B) starting from initial base content values such that the corresponding *C_GC_* and *C*_*AT*_ skews are any of the following combinations: (1, 1), (–1, 1), (1, –1) and (–1, –1). However, the subsequent results were the same as starting from initial base contents at equal proportions of 0.25 each. We ran the simulation four times separately by randomly drawing the mutation rate constants from a uniform distribution. To allow for a broad range for our random rate constants, we took the value of the symmetry-corrected maximum mutation rate (${k}_{C \to T}/{k}_{G \to A}$) observed in the human genome from (24), added its standard deviation additionally scaled by multipliers 1, 2, 5 and 10. This resulted in [0, 1.799], [0, 2.490], [0, 4.561] and [0, 8.013] ranges, for all the multipliers respectively. The discussion below operates on the outcomes with the multiplier 1, but we verified that the results did not differ much for the other tried cases. We did this for the following reason: while simulating the cases and allowing variation in the mutation rate constants, one approach would be to take the human rate constant values and fluctuate around them. However, as we do not know what kind of fluctuation is relevant in the original life, it is better to simulate cases where we go beyond a certain range, hence scaling the random sampling of the mutation rate constants.

In this most general case, all 12 rate constants are unique, and the corresponding system is numerically evolved using the following four kinetic equations:


(1) $$\begin{equation*}\begin{array}{l} \displaystyle\frac{{d{C}_A}}{{dt}} = {k}_{C \to A}\ {C}_C + {k}_{T \to A}{C}_T + {k}_{G \to A}{C}_G - \left( {{k}_{A \to C\ } + {k}_{A \to T} + {k}_{A \to G}} \right){C}_A\\[10pt] \displaystyle\frac{{d{C}_G}}{{dt}} = {k}_{A \to G}\ {C}_A + {k}_{C \to G}{C}_C + {k}_{T \to G}{C}_T - \left( {{k}_{G \to A\ } + {k}_{G \to T} + {k}_{G \to C}} \right){C}_G\\[10pt] \displaystyle\frac{{d{C}_T}}{{dt}} = {k}_{A \to T}\ {C}_A + {k}_{G \to T}{C}_G + {k}_{C \to T}{C}_C - \left( {{k}_{T \to A\ } + {k}_{T \to C} + {k}_{T \to G}} \right){C}_T\\[10pt] \displaystyle\frac{{d{C}_C}}{{dt}} = {k}_{A \to C}\ {C}_A + {k}_{T \to C}{C}_T + {k}_{G \to C}{C}_G - \left( {{k}_{C \to A\ } + {k}_{C \to T} + {k}_{C \to G}} \right){C}_C \end{array}\end{equation*}$$

where *C_s_* is the content of the base *S*, and ${k}_{s \to u}$ is the mutation rate constant for the $s \to u$ mutation. Recording the base contents at the end of the 4.28 byr period, we can see that the final *C_GC_* and *C_AT_* skews have a wide range of distribution, peaking at 0 (Figure 1C). That preference towards 0 is, however, far from the observed PR-2 compliance in actual genomes, where the compliance region is much narrower at around the 0 value for the skews. This is demonstrated in Figure 1D, where the centre of the plot is zoomed in with the compliance regions highlighted for eukaryotes, prokaryotes and DNA viruses.

Two other scenarios were also tried for the simulations, where every independent mutation rate constant is assumed to have (i) a truncated normal distribution centred at their corresponding average values for *Homo sapiens*, and (ii) symmetry-constrained rates from a truncated normal distribution centred at symmetry-averaged rates for *Homo sapiens*. The values for the mutation rates were taken from (24) and summarised in Table 1. For each of the four separate simulations, the standard deviation values were scaled by $m \in \{ {1,\ 2,\ 5,\ 10} \}$ multiplier values to examine the various spread scenarios around the averages.

**Table 1.** Rate constants used in the simulations for *i → j* mutations. There, the notation such as AC denotes a rate for the *A → T* mutation. The Individual singleton rate constants used in the simulation (A) are brought in the upper part. Those are followed by the strand symmetry-averaged singleton rates used in the simulation (B). Mutation rate constants were taken from (24)

Mutation	Mean	SD
**AC**	0.198	0.100
**TG**	0.199	0.098
**AG**	0.679	0.553
**TC**	0.875	0.824
**AT**	0.210	0.111
**TA**	0.216	0.132
**CA**	0.371	0.505
**GT**	0.269	0.139
**CG**	0.304	0.158
**GC**	0.238	0.117
**CT**	1.173	0.894
**GA**	1.044	0.387

**AC/TG**	0.198	0.099
**AG/TC**	0.753	0.673
**AT/TA**	0.212	0.119
**CA/GT**	0.322	0.378
**CG/GC**	0.273	0.144
**CT/GA**	1.109	0.690

These results are summarised in Figures S3 and S4. Interestingly, recording the final *C_GC_* and *C_AT_* skews at the end of the 4.28 byr period for the above two scenarios A) and B), there appears to be an overall, general skew observed. To the best of our knowledge, this is most likely an artefact of using a truncated normal distribution, where the sampling has an intrinsic slight positive skewness. As mutation rate constants are positive real numbers and are mostly distributed between zero and one, we could not use the normal distribution. Instead, the truncated normal distribution made the most sense for the overall shape of the distribution to resemble the bell-shaped curve with as little skew as close as possible. We, therefore, do not believe the skewness observed in Figures S3 and S4 is driven by a biological phenomenon. We also note that, at 4.28 byr time point, the solutions to the simulation with the symmetry-constrained rates B) exhibit a distinct difference in behaviour, as compared to the simulations in A). The simulation B) reveals preferential islands in *C_GC_* versus*C_AT_* skew plots (Supplementary Figure S4), and a diagonal spread that is scaled as we increase the standard deviation values for the mutation rate sampling range. Interestingly, the diagonal pattern is found in the actual skew plot for prokaryotic organisms (Figure 1A).

### *De novo* link between the unconstrained mutation rates and PR-2

**Figure 2.**
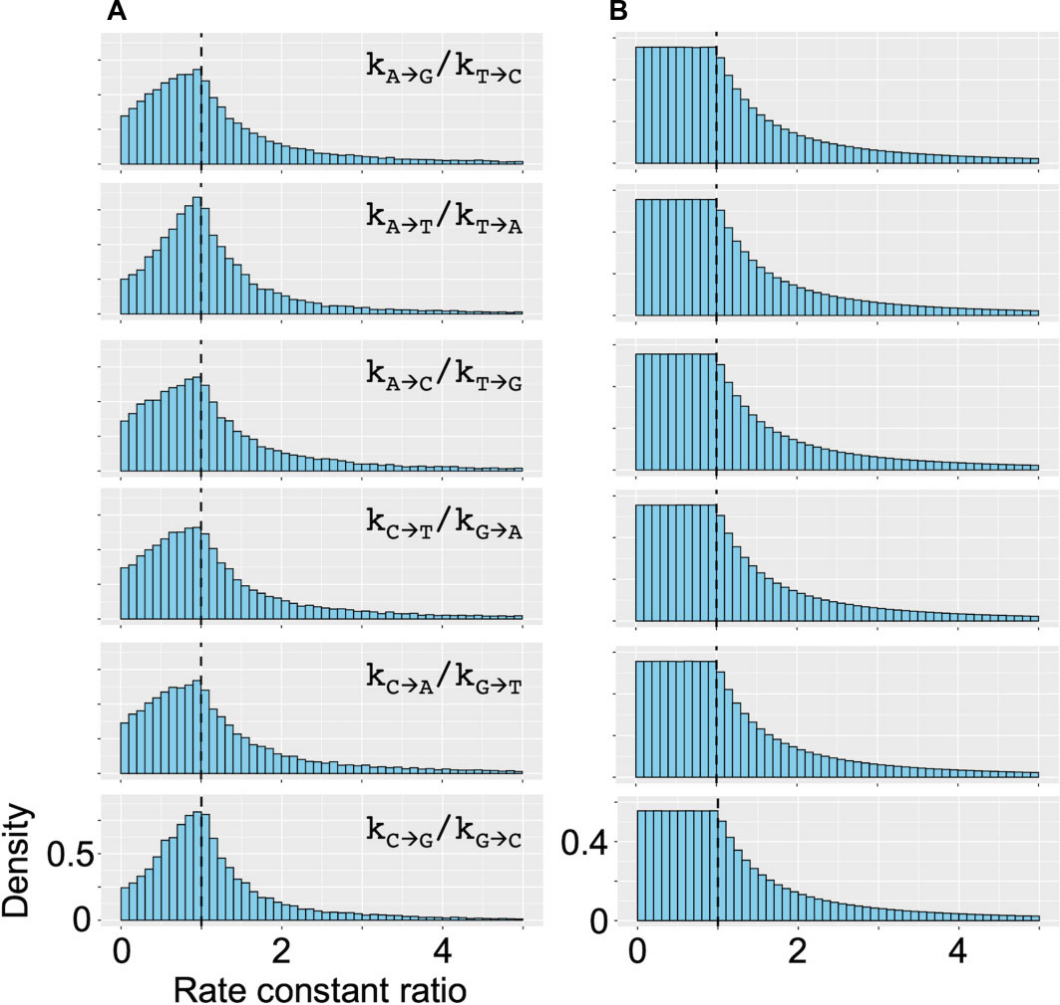
Distributions of the rate constant ratios from the numerical analysis of the unconstrained mutational network model. This represents the most general model, where all the 12 ${k}_{s \to u}$ rate constants (Figure 1A) are unique. The system is numerically solved to produce the time evolution of genomic base content within a 4.28 byr period, the current maximum estimate of the age of life on Earth (36). Plots (**A**) and (**B**) show the distributions of the ratios of ${k}_{s \to u}$ pairs that are expected to be equal (Eqs. 2) according to the no-strand-bias (NSB) assumption, but not pre-set as equal in the simulations. The set of histograms in (A) show such ratios for all the rate combinations in the PR-2 compliance zone peak at the value of 1, showing that the solutions tend to the compliance with the parity rule when the rate constants tend to satisfy the equalities stemming from the NSB assumption, though also showing a substantial variation. The set of histograms in (B) show such ratios for all the rate combinations except those in (A), the compliance zone, and have the classical ratio distribution shape, which can be obtained by dividing two uniform distributions.

For the 25 million *in silico* generated systems (section “Assumption-free approach to look for a link between mutation rates and PR-2” of Results and Discussion), we obtained the combinations of rate constants that resulted in PR-2 compliant base contents after 4.28 byr. Examining the *C_GC_* and *C_AT_* skews at the end of the simulations (Figure 1C), we can zoom into the area that is comparable to the selected PR-2 compliance regions, as inferred from the experimental genomes (Figure 1A) defined in section “The universality of PR-2 in dsDNA genomes of different species” of Results and Discussion. Figure 2 shows the ratios of ${k}_{s \to u}$ pairs that are expected to be equal under the NSB assumption, but not pre-set as equal in these simulations. We show the distributions of the ratios of ${k}_{s \to u}$ pairs for all the generated combinations of rate constants in Figure 2A that end up with the system complying with PR-2. Those in Figure 2B show a classical ratio distribution shape heralding the division of two uniform distributions (Figure 2B). Zooming onto the strict PR-2 compliance region and investigating the rate constants that resulted in low *C_GC_* and *C_AT_* skews in our simulations, the above-defined rate constant ratios become distributed differently, peaking at the value of 1, though still showing a significant spread (Figure 2A). We observe the same behaviour with two separate simulations, where A) the initial $\{ {A,T,G,C} \}$ contents are sampled within a range of allowed base content values in eukaryotic and prokaryotic organisms for the first two randomly sampled bases, with the remaining two bases being sampled from $1 - remainder$ for the four bases to always sum to 1 for a complete genome, and B) the initial base content values are sampled such that the corresponding *C_GC_* and *C_AT_* skews are any of the following combinations: (1, 1), (–1, 1), (1, –1) and (–1, –1). Therefore, with no constraint on any interdependence amongst the mutation rates (inter-mutation network brought in Figure 1B), and a wide variety of initial base compositions tried, we can see that the combinations of those 12 independent rate constants (Eqs. 1) that allow for a PR-2 compliance reflect, at least in part, the NSB rate constant equalities (Eqs. 2) within the 4.28 byr evolution. The combinations still allow a significant variation at around those equalities (Figure 2A), potentially governed by a higher degree of interdependences in mutation rates that we investigated in further sections.

### Link between the mutation rates and PR-2 under the no-strand-bias assumption

**Figure 3.**
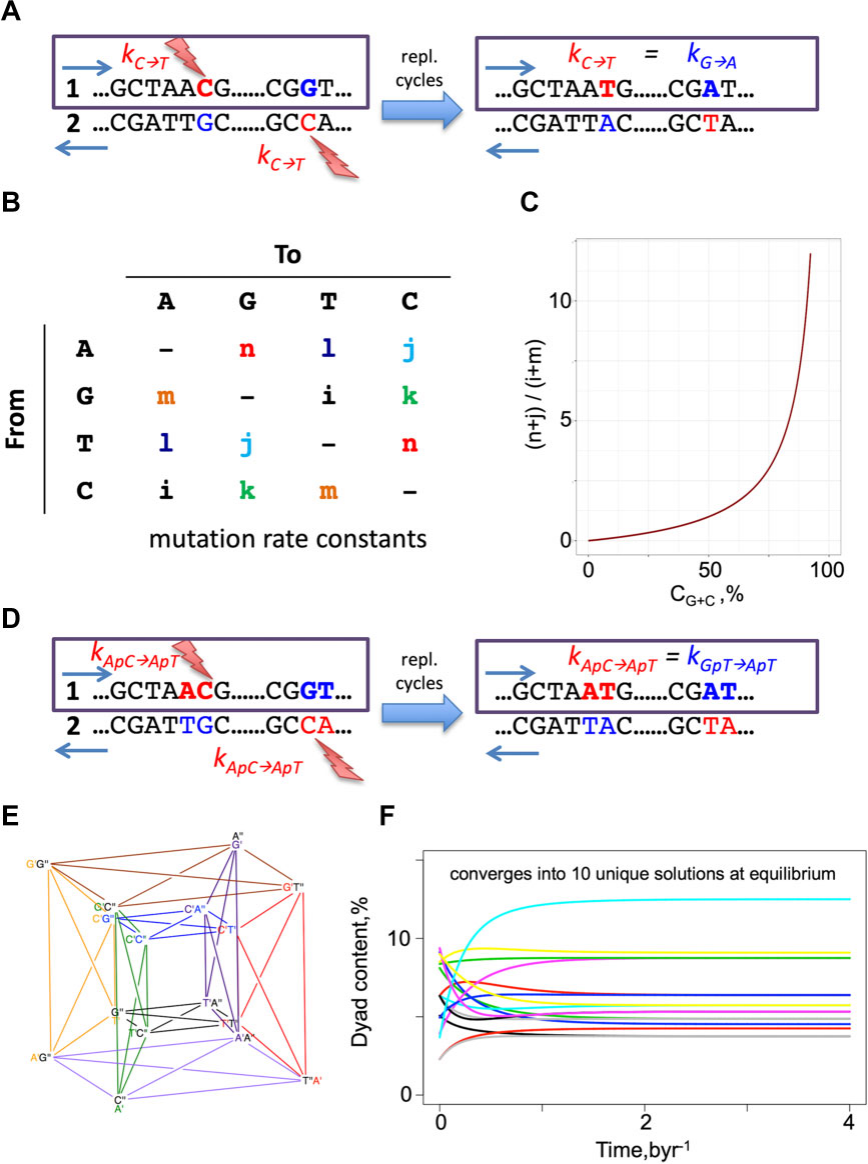
The no-strand-bias (NSB) assumption for singletons and dyads. (**A**) Illustrates the NSB principle, on the example of $C \to T$ transition. (**B**) The mutation rate matrix with only 6 independent parameters. The inferred dependency between the genomic equilibrium G+C base content and mutation rate constants is shown in (**C**). (**D**) Example of the NSB model in a dyad context. In the oligomeric context, NSB happens where both the source and the substituted states of two mutations are reverse complementary to each other. The figure demonstrates this, analogous to the single-base case discussed before, on the example of the equalities between the rates of $ApC \to ApT$ and $GpT \to ApT$ dyad mutation rates. (**E**) The schematic representation of the cross-mutation network among dyads is constructed based on the tesseract (hypercube). There, the primed superscripts denote the position in the dyad, from 5’ to 3’ direction (A’G” is the same as ApG). Each line should be interpreted as a set of two counter-directed arrows. (**F**) shows an example of the time evolution of dyad contents starting from an arbitrary set of initial dyad contents and rate constant values. The colouring scheme is random.

From section “*Denovo* link between the unconstrained mutation rates and PR-2” of Results and Discussion and Figure 2A, we inferred that there is a wide range of combinations in mutation rates that can result in genomes that exert Chargaff's PR-2 as a phenomenon emergent from those rate constants within the timeframe of the age of life on Earth. However, a part of the PR-2 compliant rate constant combinations, springing from our unconstrained simulations, showed rate equalities similar to the NSB assumption. Therefore, before going on to investigate the more generalised rules that may exist to link mutation rates with PR-2 compliance, here we first outline the simpler model under the NSB assumption. As discussed in prior literature, equalities of certain mutation rates could be present in DNA owing to its complementary double-stranded nature, if we assume NSB for mutation rates (6,14). To clarify how the mutation rate equalities emerge, consider the example of ${k}_{C \to T} = {k}_{G \to A}$ (Figure 3A). Central to the NSB model is a plausible assumption of the strand-invariance of the mutation rates, i.e. the $C \to T$ mutation happens at the same rate independently from whether $C$ is in the template or complementary strand of the dsDNA. Therefore, each strand will also have the complementary $G$ to $A$ conversions with a rate similar to $C$ to $T$ conversion, hence ${k}_{C \to T} = {k}_{G \to A}$. This symmetry in rate constants significantly simplifies the mutation network from 12 to 6 independent rate constants:


(2) $$\begin{equation*}\begin{array}{l} {k}_{C \to A} = {k}_{G \to T}\ = \ i\\ {k}_{A \to C} = {k}_{T \to G}\ = \ j\\ {k}_{C \to G} = {k}_{G \to C}\ = \ k\\ {k}_{A \to T} = {k}_{T \to A}\ = \ l\\ {k}_{C \to T} = {k}_{G \to A}\ = \ m\\ {k}_{A \to G} = {k}_{T \to C}\ = \ n \end{array}\end{equation*}$$

The reduced number of independent rate constants from the NSB assumption produces a simpler mutation matrix (Figure 3B), and a simpler system of kinetic equations:


(3) $$\begin{equation*}\begin{array}{l} \displaystyle\frac{{d{C}_A}}{{dt}} = i{C}_C\ + l{C}_T + m{C}_G - \left( {j + l + n} \right){C}_A\\[10pt] \displaystyle\frac{{d{C}_G}}{{dt}} = n{C}_A\ + k{C}_C + j{C}_T - \left( {m + i + k} \right){C}_G\\[10pt] \displaystyle\frac{{d{C}_T}}{{dt}} = l{C}_A\ + i{C}_G + m{C}_C - \left( {l + n + j} \right){C}_T\\[10pt] \displaystyle\frac{{d{C}_C}}{{dt}} = j{C}_A\ + n{C}_T + k{C}_G - \left( {i + m + k} \right){C}_C \end{array}\end{equation*}$$

each describing the evolution of A, G, T and C base contents in fractions. At equilibrium, the system is fully solvable, and the equilibrium base contents are given through the following equations (see Supplementary Note S1 for the derivation):


(4) $$\begin{equation*}\begin{array}{l} {C}_A = {C}_T\ = \displaystyle\frac{{\left( {i + m} \right)}}{{2\left( {i + j + m + n} \right)}}\\[10pt] {C}_G = {C}_C\ = \displaystyle\frac{{\left( {j + n} \right)}}{{2\left( {i + j + m + n} \right)}} \end{array}\end{equation*}$$

The solutions imply the ${C}_A = {C}_T$ and ${C}_G = {C}_C$ equalities for base composition at equilibrium under NSB for mutation rates. These solutions also link the mutation rate constants (molecular level characteristics of a DNA) with the equilibrium genome composition. We can express that link for the overall G+C content of any genome (under NSB at equilibrium) as follows (Supplementary Note S1):


(5) $$\begin{equation*}\frac{{n + j}}{{i + m}} = \frac{{{C}_{G + C}}}{{\left( {1 - {C}_{G + C}} \right)}}\ \end{equation*}$$

where the G+C content is naturally independent from ${k}_{C \to G} = {k}_{G \to C}$ and ${k}_{A \to T} = {k}_{T \to A}$ mutation rate constants. This results in a plot in Figure 3C, which shows where the G+C contents of different genomes should lie under the NSB assumption at equilibrium.

The NSB assumption can lead to significant simplifications also when considering oligomers of size >1 for cross-mutations. For instance, the dyad case inter-mutational network can be represented by a tesseract (hypercube), with additional connections within, where each edge represents a mutation in one of the two bases between the dyad of one node to the dyad of another node (Figure 3C). We can see this in the example of the $ApC \to ApT$ mutation in Figure 3D. Under the NSB assumption, the system of 16 kinetic equations consisting of 96 independent mutation rate constants can be reduced into 48 mutation rate constants. The solutions show that this system, independently from the initial rate constant of base composition values, always equilibrates into a set of 10 unique solutions for the base content (Figure 3F, full derivation in Supplementary Note S2). Those 10 solutions, indeed, reflect PR-2, as follows:


$$\begin{equation*}\left[ {{C}_{ApA} = {C}_{TpT}\ } \right],\ \left[ {{C}_{ApC} = {C}_{GpT}\ } \right],\ \left[ {{C}_{ApG} = {C}_{CpT}\ } \right],\ \left[ {{C}_{CpA} = {C}_{TpG}\ } \right],\end{equation*}$$

(6) $$\begin{equation*}\left[ {{C}_{GpA} = {C}_{TpC}\ } \right],\ \left[ {{C}_{CpC} = {C}_{GpG}\ } \right],\ {C}_{CpG},\ {C}_{GpC},\ {C}_{ApT},\ {C}_{TpA}\end{equation*}$$

In an even simpler case, where we assume context independence of the mutation rates, the number of the independent rate constants can further go down into three unique solutions at equilibrium (analytical solutions found in Supplementary Note S2).


$$\begin{equation*}\left[ {{C}_{ApA} = {C}_{TpT}\ } \right] = \left[ {{C}_{ApT}} \right]\ = \left[ {{C}_{TpA}} \right]\ = \frac{{{{\left( {i + m} \right)}}^2}}{{4{{\left( {i + j + m + n} \right)}}^2}} \end{equation*}$$

$$\begin{eqnarray*}[ {{C}_{ApC} = {C}_{GpT} } ] = [ {{C}_{ApG} = {C}_{CpT}} ] = [ {{C}_{CpA} = {C}_{TpG}} ] = [ {{C}_{GpA} = {C}_{TpC}} ] = \frac{{( {i + m} )( {j + n} )}}{{4{{( {i + j + m + n} )}}^2}} \end{eqnarray*}$$

(7) $$\begin{equation*}\left[ {{C}_{CpC} = {C}_{GpG}\ } \right] = \left[ {{C}_{CpG}} \right]\ = \left[ {{C}_{GpC}} \right]\ = \frac{{{{\left( {j + n} \right)}}^2}}{{4{{\left( {i + j + m + n} \right)}}^2}} \end{equation*}$$

The above expressions reflect the connection between the genomic dyad content and the underlying individual rate constants. As expected, the three equilibrium solutions from dyad NSB, without context dependence of mutation rates, are the cross multiplications of the two unique equilibrium solutions obtained from singleton NSB, which is what is dictated by probability theory for the co-occurrence of independent events. Thus, by using the principle of mathematical induction, there is no clear reason why we cannot extend this model to higher k-mers too.

### The plausibility of NSB assumption in actual genomes

**Figure 4.**
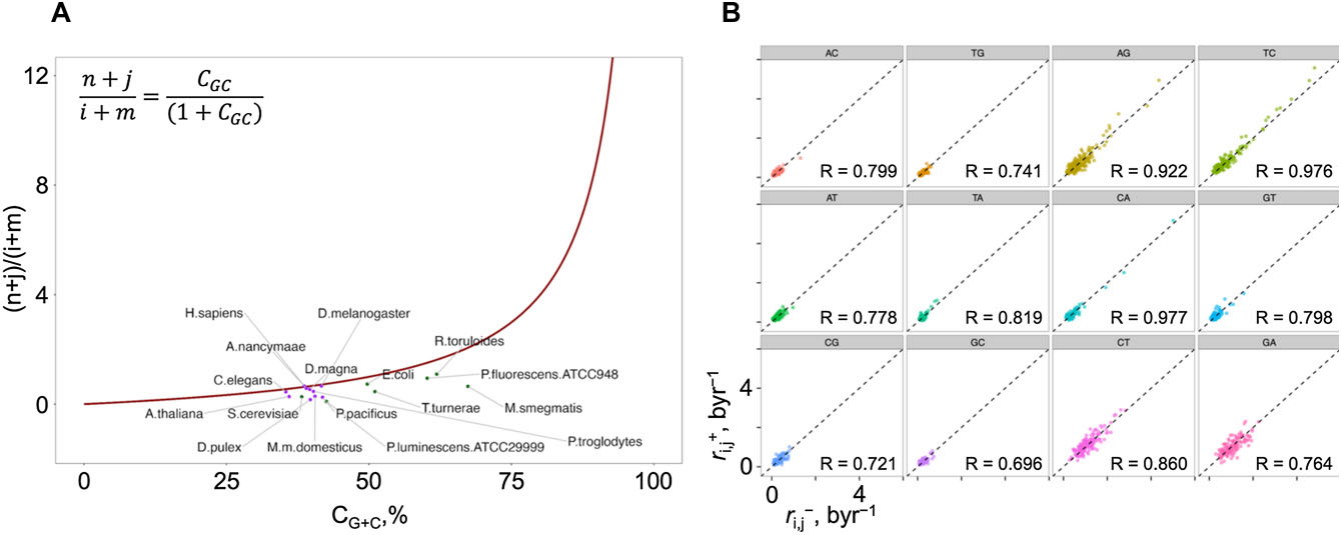
Strand-wise average mutation rate constants in humans. (**A**) The link between the mutation rates and the G+C content at equilibrium, along with the actual values for observed genomes (full details in Supplementary Figure S5). (**B**) 12 sets of mutation rate constants, averaged across the length of the human genome, for each, in only + and in only -, strand of the dsDNA, obtained using the Trek methodology (22). The plots show the correlation between the 7-meric mutation rates in + and - strands, where each point represents one 7-mer with its central 4^th^ base undergoing the mutation of the plot category. The Pearson correlation coefficient values are indicated on each plot, where the ${k}_{C \to A}$ showed the highest value (*R* = 0.977).

As reviewed above, no strand bias (NSB) in the mutation rate constants (6,14) may become in conflict with experimental observations, as any deviation of the mutation rates from NSB, and/or genomic compositions from PR-2 would suggest that the model is wrong or that the system is out of equilibrium. In this section, we show that the NSB model can, overall, be considered a good reductionist assumption for genomes, and highlight that there are indeed variations at a more regional scale and for some of our tested genomes. In section “Link between the mutation rates and PR-2 under the no-strand-bias assumption” of Results and Discussion, we showed that, under the NSB assumption, base compositional equalities at equilibrium are achieved and showed how this is linked with the overall G+C content of any genome. This results in a plot with interesting behaviour shown in Figure 3C. To investigate how close the present-day genomes lie along this ideal curve, we obtained the strand symmetric mutation rate constants from 17 species across the eukaryotic and prokaryotic kingdoms along with their G+C content and overlaid it with the curve in Figure 4A (25,37–46). On average, the G+C content of the 10 eukaryotes deviates away by approximately 10% and 18% for the 7 prokaryotes. Interestingly, for all of the 17 species, except for *Aotus nancymaae*, the observed mutation rate constants suggest that the true G+C content should, in fact, be lower than what we observe in the present day. This may suggest that the genome composition is yet to reach the full equilibrium under NSB. Indeed, as we shall explain in further detail in section “General implications for life: how long does it take to converge to PR-2?” of Results and Discussion, the NSB model suggests that the average time to reach PR-2 compliance occurs within 4.28 byr, the current maximum estimate for the age of life on Earth (36). However, using the strand symmetric mutation rate constants for 6 of the 10 eukaryotes to numerically calculate the equilibrium base content, we find that *Escherichia coli*, *Caenorhabditis elegans*, *Drosophila melanogaster* and *Homo sapiens* should be closer to or already reaching PR-2 compliance under NSB, while *Aotus thaliana* and *Saccharomyces cerevisiae* are expected to achieve equilibrium much later (Supplementary Figure S7). These results broadly match the deviation of the G+C content between the theoretical value at equilibrium and the true value. To investigate the plausibility of NSB assumption further, we employed the Trek methodology (24), in which we studied the 7-meric context-dependent germline mutation rates for the human genome. We found that the 12 sets of mutation rate constants for each of the plus and minus strands of the dsDNA, still averaged across the whole genomic span for each strand, are, in fact, highly correlated for the human genome (Figure 4B). This indicates that, while allowing for a regional deviation, NSB holds true for the average mutation rates summarised across the wider length of the human genome.

### A generalised link between mutation rates and PR-2

Since the prior considerations and our assumption-free simulation (section “De novo link between the unconstrained mutation rates and PR-2” of Results and Discussion) show a substantial variation of the mutation rates that can result in PR-2 while not conforming NSB (Figure 2), here, we tried to see whether a more generalised model can be found that can predict the PR-2 compliance of a genome, within 4.28 byr of evolution, not necessarily at compositional equilibrium, from mutation rates that are not necessarily NSB compliant. We used a symbolic regression modelling engine, Eureqa (now part of DataRobot), to find interrelations between the constituent 12 mutation rate constants from the sets under the tolerance region of the PR-2 compliance (47,48). Eureqa, developed by Schmidt *et al.*, determines the functional relationships to best describe the dataset in the simplest form *via* evolutionary search. The algorithm starts out with a random initial population of some unary (trigonometric, exponential, etc.) and binary (addition, subtraction, etc.) operators that collectively make up the tree-based graph. Next, evolutionary algorithm mechanisms take place, including mutation and crossover, each with an associated probability, to create offspring. A fitness score is evaluated (mean squared error) on these newly created offspring. The fittest offspring will be selected for reproduction, which, in symbolic regression, is evaluated by which functional forms describe the data better than others. The least-fit offspring will be replaced by a new population and this process repeats.

Each mutation rate constant was evaluated as a function of the remaining 11 mutation rate constants in the PR-2 compliance zone, which revealed more general mathematical dependencies that are more relaxed than the simple equalities of mutation rates under the NSB assumption. Our new set of generalised mutation rate constraints can be described through sets of equations comprised of simple linear combinations of interchangeable mutation rate constants. For each mutation rate constant, Eureqa generated several equations, but we selected the ones that strike a balance between low error (most accurate) and complexity (measured by the size and mathematical complexity of the symbolic expression). At times, the symbolic expressions could reach a high complexity, thus making it difficult to understand the interrelations between the mutation rate constants in a simple manner. We found that a complexity in the range of 7-9 terms provided this simplicity yet maintained its high accuracy. The found 12 simplified constraint equations are as follows:


(8) $$\begin{equation*}\begin{array}{l} {k}_{ag} = \ - 0.6\left( {kcg - kgc + kat - kta + kct - 1.3ktc - kga - 0.6} \right)\\ {k}_{ga} = \ 0.7\left( {kcg - kgc + kat - kta + kct - ktc + kag + 0.1} \right)\\ {k}_{tc} = \ 0.8\left( {0.7kcg - kgc + 0.8kat - kta + kag - 0.8kga + kct + 0.4} \right)\\ {k}_{ct} = \ - 0.8\left( {kcg - kgc + 0.8kat - kta + kag - kga + 0.1kca - ktc - 0.3} \right)\\ {k}_{ac} = \ - 0.8\left( {kgc - kcg + kat - kta + kgt - ktg - kca - 0.3} \right)\\ {k}_{ca} = \ 0.8\left( {kgc - kcg + kat - kta + kgt - ktg + kac + 0.2} \right)\\ {k}_{gt} = \ - 0.8\left( {kgc - kcg + kat - kta - ktg + kac - kca - 0.2} \right)\\ {k}_{tg} = \ 0.8\left( {kgc - kcg + kat - kta + kgt + kac - kca + 0.1} \right)\\ {k}_{at} = \ - 0.5\left( { - 2kta + kct - ktc + kag - kga + kgt - ktg + kac - kca - 0.5} \right)\\ {k}_{ta} = \ 0.5\left( { + 2kat + kct - ktc + kag - kga + kgt - ktg + kac - kca - 0.1} \right)\\ {k}_{gc} = \ - 0.4\left( { - 2kcg + ktc - kct + kga - kag + kgt - ktg + kac - kca - 0.06} \right)\\ {k}_{cg} = \ 0.4\left( {2kgc + ktc - kct + kga - kag + kgt - ktg + kac - kca + 0.9} \right)\end{array}\end{equation*}$$

We can thus state that any mutation rate combination that complies with the above set of equations will lead to a PR-2 compliant genome. To test the performance of the 12 equations, we repeated the simulation of replicates following a non-symmetric, uniform distribution (equations in Figure 1B) by generating 10 million systems and applying the same PR-2 tolerance values from eukaryotic organisms. Using the test set on the 12 equations, we reveal that all equations show a strong, positive correlation between the true and predicted value (Supplementary Figure S6). This shows that the equations are, on average, able to capture the majority of the mathematical dependencies amongst the mutation rates that result in PR-2 genomes with high accuracy.

**Figure 5.**
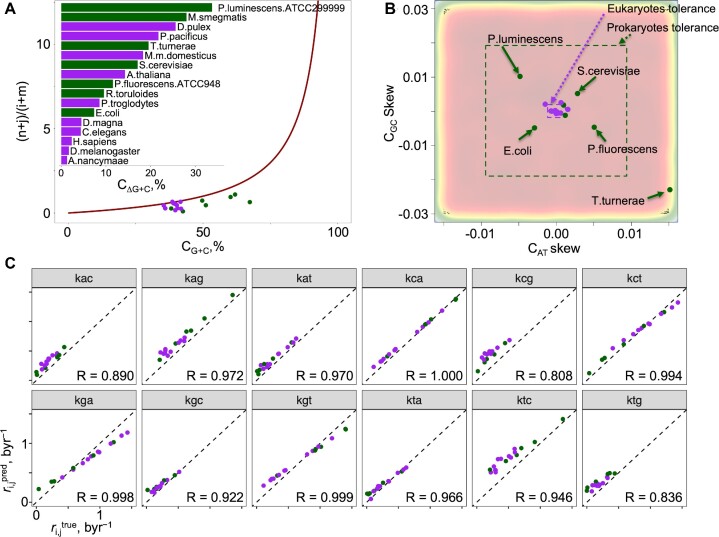
Comparison between species in terms of their closeness to NSB-driven equilibrium and closeness to compliance to our generalised equations. (**A**) The base content solutions at equilibrium reveal the dependencies between the genomic G+C content and the mutation rate constants (red line). The strand symmetric mutation rate constants were obtained from 17 species across the eukaryotic and prokaryotic kingdoms (25,37–46) and overlaid as a scatterplot with colourings based on their associated eukaryotic (purple) or prokaryotic (dark green) kingdoms. The difference in the G+C content between the true value and the theoretical value along the red curve was calculated for each species, coloured by kingdom, and represented as a bar plot within, sorted in decreasing order: *Photorhabdus luminescens ATCC29999, Mycobacterium smegmatis, Daphnia pulex, Pristionchus pacificus, Teredinibacter turnerae, Mus musculus domesticus, Saccharomyces cerevisiae, Arabidopsis thaliana, Pseudomonas fluorescens ATCC948, Rhodosporidium toruloides, Pan troglodytes, Escherichia coli, Daphnia magna, Caenorhabditis elegans, Homo sapiens, Drosophila melanogaster, Aotus nancymaae*. (**B**) The four systems of equations numerically solved for 25 million systems based on the most general model of 12 independent mutation rate constants randomly drawn from a uniform distribution based on values obtained from the Trek methodology (24) in byr range. Recording the base content *C_GC_vs. C_AT_* skew at the final 4.28 byr time point for all systems and zooming in the *C_AT_* skew range of ±0.015 and *C_GC_* skew range of ±0.03, the 2-dimensional kernel density estimate scatterplot in (B) is obtained. The same 17 species from (A) are overlaid and coloured in the same scheme as in (A). The dotted boxes represent the PR-2 compliant zones for the eukaryotic (purple) and prokaryotic (dark green) kingdoms, where each edge of the box represents the total $\bar{x} \pm s$ range for the corresponding axis (see main paper for exact values). (**C**) The strand symmetric mutation rate constants obtained from the 17 species across the two kingdoms, aligned to the Trek-scaling (24) and substituted the parameters of the 12 sets of mutation rate constant equations. Each plot represents one of the 12 mutation rate constants where each point represents a species, coloured by their corresponding kingdom in the same colouring scheme as in (A) (Supplementary Figure S8 shows all 12 mutation rate constants in each plot, where any given plot is one of 17 species). The Pearson correlation coefficient values are indicated on each graph, where the diagonal dotted line represents perfect correlation with a slope of 1.

As initially discussed in section “The plausibility of NSB assumption in actual genomes” of Results and Discussion, to examine how close the current PR-2 compliant lifeforms are to the fully equilibrated NSB solution (Figure 5A), we took the mutation rate constants of various species from the eukaryotic and prokaryotic kingdoms (25,37–46) with their associated genomic G+C content and compared it to their expected NSB dependency at equilibrium (the theoretical line). We found that the majority of eukaryotic species are closer to the full equilibrium NSB solution, while prokaryotic species tend to be further away, despite all species falling within the PR-2 tolerance region (Figure 5B). Therefore, we can outline the presence of genomes that are far from NSB/equilibrium but are still PR-2 compliant. If we apply our set of 12 generalised constraints (Eqs. 8), we can verify that the mutation rates of such genomes are still within the relations to make a PR-2 compliant genome within 4.28 byr. Importantly, we believe this demonstration confirms that the drivers of PR-2 are still mutation rates, though with constraints more permissive and deviant from the previously proposed stricter NSB equalities. To convert the mutation frequencies into the genomic average mutation rate constants in a time domain (Trek-scaling), we used the equation $k = 2.831f$ (see Materials and Methods for details on obtaining this equation), where $f$ is the mutation frequency for a given mutation ${k}_{s \to u}$ for a given species. The resulting mutation rate constants were passed through the generalised equations above (Eqs. 8). Here, we demonstrate that the relations are linear and highly correlated (Figure 5C) for all the 12 constraint equations, more generalised than the simpler NSB equalities. To reiterate, Eqs. 8 are obtained from a simulated system with no assumption and prior constraints on the mutation rates in our source simulations, but purely on the PR-2 compliance of the state after 4.28 byr of evolving. These equations are then applied to PR-2 compliant species with known mutation rate constants. Despite some of those species being out of the NSB-driven equilibrium, our relaxed equations still predict their PR-2 compliance. This reinstates the early view that behind PR-2 are the mutation rates, which we now show do not necessarily need to comply with NSB, as soon as they conform to our more generalised constraints that seem to be in place for all the tried PR-2 compliant species.

The generalised equations (Eqs. 8) revealed above for each of the 12 mutation rate constants were evaluated as a function of the remaining 11 rate constants in the PR-2 compliance zone. Still focusing on the complete spectrum of mutation rate constant repertoire, we next explored a different approach. Can we have a machine learning model that can predict the PR-2 compliance from only the initial rate constants, without numerically solving the ODE system? To this end, we developed a machine learning model to classify PR-2 compliance as a function of the 12 independent, uniform distributed mutation rate constants (section “Assumption-free approach to look for a link between mutation rates and PR-2” of Results and Discussion). The model revealed that the four mutation rate constants ${k}_{A \to T},\ {k}_{T \to A},\ {k}_{G \to C},$ and ${k}_{C \to G}$ were the most important for classifying PR-2 compliance (see Supplementary Note S2 for details).

### General implications for life: how long does it take to converge to PR-2?

Developing our simulations towards targeting more generalised characteristics of life, here we seek to explore the following three questions. (i) What is the average time it takes a genome to reach PR-2 compliance? (ii) What is the average time it takes a genome to reach a compositional equilibrium? (iii) Are there significant differences between the times of (i) and (ii)? To do this, we analysed the kinetic system of equations by starting from random $\{ {A,T,G,C} \}$ contents within the range of allowed base content values in eukaryotic and prokaryotic organisms for the first two randomly sampled bases, with the remaining two bases being sampled from $1 - remainder$ for the four bases to always sum to 1 for a complete genome, with symmetry-constrained mutation rate constants randomly drawn from a normal distribution. Previously, all simulations started with the initial base content of 25% for each of the four bases, however, in the following work, we remove this restriction as we seek to explain the universal nature of PR-2 from a species-invariant perspective. We used the PR-2 tolerance values obtained from the prokaryotic organisms because we learned from the simulation work that the number of systems that fall within the PR-2 compliance zone using the eukaryotic organisms is too small of a fraction from which we could draw any significant conclusions. We can, however, circumvent this problem by using the PR-2 tolerance values from the prokaryotic organisms instead and we also know that these species comply with PR-2.

**Figure 6.**
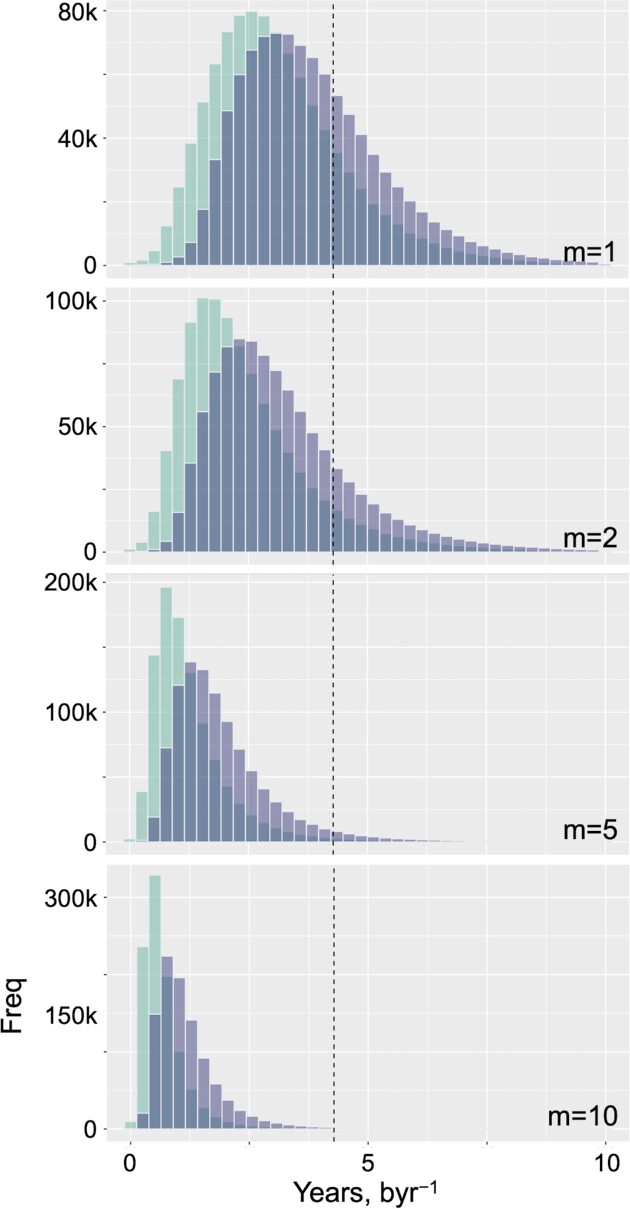
Distribution of time for a genome to reach PR-2 compliance and compositional equilibration. 10 million systems with the simulation model were generated where the initial base content was randomly sampled from the maximum allowed range based on eukaryotic and prokaryotic organisms. The strand symmetric mutation rate constants were randomly drawn from a truncated normal distribution. This process was performed four separate times, each with a different multiplier, $m$, applied to the standard deviation of the random sampling of the mutation rate constants $m\ \epsilon \{ {1,2,5,10} \}$. The green histograms represent the distribution of time to reach PR-2 compliance and the purple histograms represent the distribution of time to reach genome equilibrium (see Materials and Methods for details). The vertical line intercepts the x-axis at 4.28 billion years, the maximum current estimate of the age of life on Earth (36).

Performing this simulation, we arrive at an average time to reach PR-2 compliance well within 4.28 byr (Figure 6), the current maximum estimate for the age of life on Earth (36). This happens independently from the initial genome composition and from the randomly selected wide range of mutation rates at around their human genome values. Interestingly, the system can start to comply with PR-2 even before reaching the base content equilibrium. In fact, on average, the PR-2 compliance reaches approximately one byr before the time to reach compositional equilibration (see Materials and Methods for PR-2 tolerance and genome equilibrium definitions). The average time to reach PR-2 compliance and genome equilibration becomes earlier as we increase the scaling factor. This may be explained in the following way: as we increase the scaling factor, we independently pick from a wider range of values from which one can randomly draw the mutation rate constants. As we do not consider the inter-dependence of the different mutation rate constants, we naturally arrive at a situation where the difference between the forward and reverse rate of reaction is greater, thereby arriving at an equilibration faster. These observations explain the species-invariant and universal nature of PR-2, as all the genomes have had enough time to reach compliance.

To compare this emergent behaviour to extant genomes, we obtained the strand-symmetry-accounted normalised mutation fractions from work done by Michael Lynch (25). These values first need to be converted to mutation rate constants in the time domain (average mutation rate per site per billion years) so we can use it in our simulation model (see Materials and Methods for details). Following conversion, we set these mutation rate constants as the mean value in the normal distribution per species. As done previously, we generate 10 million systems with the simulation model for each of the six species. *Yeast* and *Arabidopsis thaliana* take, on average, the longest time to reach equilibration of the genome and PR-2 compliance. *Caenorhabditis elegans*, *Drosophila melanogaster* and *Escherichia coli* take a similar amount of time to reach equilibration and PR-2 compliance and are all below the current maximum estimate of the age of life on Earth (Supplementary Figure S7). The difference between the time to reach PR-2 compliance and genome equilibration averages well below two byr.

Overall, our results demonstrate that by using *de novo* assumption-free simulations from kinetic equations and random human mutation rate constants, we arrive at a set of 12 equations, which if satisfied by mutation rates of a given genome, would result in a genome compliant with PR-2 even with strand biases in mutation rates and even with out-of-compositional-equilibrium state. We show that, indeed, the dsDNA-based genomes, all the ones for which we could find mutation rate data, conform to these equations. Thus, our work reinstates the mutation rates as the major drivers behind the emergence of PR-2 compliant genomes demonstrating the simple principles behind the complex question, and can serve as an important basis for future molecular evolution studies in genomics.

### Abbreviations

dsDNA, double-stranded DNA; ssDNA, single-stranded DNA; NSB, no-strand-bias; PR-1, Chargaff's first parity rule; PR-2, Chargaff's second parity rule; myr, million years; byr, billion years; SNP, single nucleotide polymorphism; XGBoost, extreme gradient boosting; ML, machine learning; AUC, the area under the curve; ROC, the receiver operating characteristic; AUROC, the area under the receiver operating characteristic curve.

## Supplementary Material

gkad477_Supplemental_File

## Data Availability

The computer code, necessary to process the species across the three kingdoms, perform the simulations, and employ the machine learning strategy to perform the remaining computations, can be accessed through the following GitHub repository: https://github.com/SahakyanLab/GenomicPR2Simulations, and the Genomic PR-2 Simulation web application is accessible on https://github.com/SahakyanLab/GenomicPR2SimsWebApp. The data and accompanying computer code are also available at Zenodo: https://doi.org/10.5281/zenodo.7688731. All the used public datasets are accessible from the established genomic data repositories as detailed in Materials and Methods.
