# Supplementary material for: Generalised interrelations among mutation rates drive the genomic compliance of Chargaff's second parity rule
Source: Nucleic Acids Res. 2023 Jun 9;51(14):7409–23. doi: 10.1093/nar/gkad477 (PMC10415130; doi:10.1093/nar/gkad477)
Supplement: gkad477_Supplemental_File [file gkad477_supplemental_file.pdf]

Generalised interrelations among mutation rates drive the genomic compliance of Chargaff's second parity rule
Supplementary Information

Patrick Pflughaupt and Aleksandr B. Sahakyan

January 23, 2023

Contents

Note S1. Derivations of the full equilibrium solutions **2**

Note S1.1. The analytic solutions of the mutation rates under no-strand-bias assumption at equilibrium . . . 2

Note S1.2. The NSB principle can be extended into higher k-meric orders . . . 3

Note S1.3. Application of the mutation rate constants under no-strand-bias in predicating singleton and dyad composition of chimpanzee genome . . . 10

Note S2. Machine learning model for classifying PR-2 compliance **12**

Supplementary Figures **15**

References **23**

Note S1. Derivations of the full equilibrium solutions

Note S1.1. The analytic solutions of the mutation rates under no-strand-bias assumption at equilibrium

Under the no-strand-bias (NSB) assumption, we can consider the example of $\mathrm{k}_{\mathrm{C}\rightarrow\mathrm{T}} = \mathrm{k}_{\mathrm{G}\rightarrow\mathrm{A}}$, where the $s\rightarrow u$ subscript denotes the mutation of the base s into u. Here, $\mathrm{k}_{\mathrm{s}\rightarrow\mathrm{u}}$ is the rate constant, which involves all processes that initiate and fixate the mutation in dsDNA, also converting the complementary strand. Central to our model is a plausible assumption of the strand-invariance of the mutation rates, i.e. the $s\rightarrow u$ substitution happens at the same rate independently from whether s is in the template or complementary strand of the dsDNA. To this end, at whichever rate C mutates to T in the template strand, with the same rate C converts to T in the complementary strand. Therefore, each strand will also have the complementary G to A conversions with the rate similar to C to T conversion, hence $\mathrm{k}_{\mathrm{C}\rightarrow\mathrm{T}} = \mathrm{k}_{\mathrm{G}\rightarrow\mathrm{A}}$. This symmetry in rate constants significantly simplifies the mutation network from 12 to six independent rate constants, as such:

$$
\begin{aligned}
k_{C\rightarrow A} &= k_{G\rightarrow T} = i \\
k_{A\rightarrow C} &= k_{T\rightarrow G} = j \\
k_{C\rightarrow G} &= k_{G\rightarrow C} = k \\
k_{A\rightarrow T} &= k_{T\rightarrow A} = l \\
k_{C\rightarrow T} &= k_{G\rightarrow A} = m \\
k_{A\rightarrow G} &= k_{T\rightarrow C} = n
\end{aligned} \tag{1}
$$

The reduced number of independent rate constants under the NSB assumption, produces a system of four kinetic equations, as such:

$$
\begin{aligned}
\frac{dC_A}{dt} &= iC_C + lC_T + mC_G - (j+l+n)C_A \\
\frac{dC_G}{dt} &= nC_A + kC_C + jC_T - (m+i+k)C_G \\
\frac{dC_T}{dt} &= lC_A + iC_G + mC_C - (l+n+j)C_T \\
\frac{dC_C}{dt} &= jC_A + nC_T + kC_G - (i+m+k)C_C
\end{aligned} \tag{2}
$$

each describing the evolution of A, G, T and C base contents in fractions, making it possible to infer the time evolution dynamics of genomic base composition using different values for the rate constants and the initial base contents. Taking into account the outlined NSB-driven equalities, we can convert the system of four ODEs into a substitution matrix, M, as follows:

$$
M^{NSB} = \begin{bmatrix} -(j+l+n) & n & l & j \\ m & -(j+l+n) & i & k \\ l & j & -(j+l+n) & n \\ i & k & m & -(j+l+n) \end{bmatrix} * \begin{bmatrix} A \\ G \\ T \\ C \end{bmatrix} = \begin{bmatrix} 0 \\ 0 \\ 0 \\ 0 \end{bmatrix} \tag{3}
$$

from which we can solve for the equilibrium base contents. In *Mathematica*, the system can be specified as:

```
DSolve[{ CA'[t]==i*CC[t]+l*CT[t]+m*CG[t]-(j+l+n)*CA[t],
         CG'[t]==n*CA[t]+k*CC[t]+j*CT[t]-(m+i+k)*CG[t],
         CT'[t]==l*CA[t]+i*CG[t]+m*CC[t]-(l+n+j)*CT[t],
```

```
            CC'[t]==j*CA[t]+n*CT[t]+k*CG[t]−(i+m+k)*CC[t],
            CA[0]==CA0,
            CG[0]==CG0,
            CT[0]==CT0,
            CC[0]==CC0},{CA,CG,CT,CC},t]//FullSimplify
```

The solution to the ODE system is, however, too complex but we can obtain the symbolic solution of the system at equilibrium. There, the base contents are supposed to stay constants, hence we need to solve the system of equations displayed below, additionally setting the sum of all the base contents (in fractions) to 1.

```
Solve[ i*CC + l*CT + m*CG − (j + l + n)*CA == 0 &&
       n*CA + k*CC + j*CT − (m + i + k)*CG == 0 &&
       l*CA + i*CG + m*CC − (l + n + j)*CT == 0 &&
       j*CA + n*CT + k*CG − (i + m + k)*CC == 0 &&
       CA + CT + CG + CC == 1,
       {CA, CG, CT, CC}] // FullSimplify
```

$$
\begin{aligned}
C_A &\to \frac{(i+m)}{2(i+j+m+n)} \\
C_T &\to \frac{(i+m)}{2(i+j+m+n)} \\
C_G &\to \frac{(j+n)}{2(i+j+m+n)} \\
C_C &\to \frac{(j+n)}{2(i+j+m+n)}
\end{aligned}
\tag{4}
$$

The system is fully solved, and the solutions imply the $\mathrm{C_A} = \mathrm{C_T}$ and $\mathrm{C_G} = \mathrm{C_C}$ equalities at equilibrium under NSB assumption for mutation rates. These solutions also link the mutation rate constants with the equilibrium genome composition. We can express that link for the overall G+C content content of any genome, under the NSB equilibrium, as follows:

$$
\frac{C_{G+C}}{C_{A+T}} = \frac{2\frac{j+n}{2(i+j+m+n)}}{2\frac{i+m}{2(i+j+m+n)}}
\tag{5}
$$

Since $C_{A+T} = 1 - C_{G+C}$,

$$
\frac{C_{G+C}}{1 - C_{G+C}} = \frac{j+n}{i+m}
\tag{6}
$$

where the G+C content content is naturally independent from $\mathrm{k_{C \to G}} = \mathrm{k_{G \to C}}$ and $\mathrm{k_{A \to T}} = \mathrm{k_{T \to A}}$ mutation rate constants.

Note S1.2. The NSB principle can be extended into higher k-meric orders

The NSB principle can lead to significant simplifications when considering oligomers of any size for cross-mutations, hence we can potentially exploit the extra information dyad-, triad- etc. counts may reflect on the genome. In section , the rate constants were reflecting the average singleton mutations across different sequence context found in a genome. Thus, the above model is applicable for finding the individual base contents in the scale of the entire genome. In contrast to this, when we want to recover the k-mer $k \in$

{1,2,3,4,...} frequencies in a given genome, we need to consider the rate constants for many more k-mer transitions, as the neighbouring bases alter the mutation rate at a given site. To clarify this, consider the $k_{CpA \rightarrow TpA}$ and $k_{CpG \rightarrow TpG}$ substitutions in the dyad context. Both substitutions are a result of a C→T mutation. However, the rates for the two substitutions will be different ($k_{CA \rightarrow TA} \neq k_{CG \rightarrow TG}$), because of the neighbouring base (A vs. G) effects. In this particular example, we know that in the CpG context, the mutation rates for C are substantially elevated [1–4]. The equalities between the rate constants noted in section are true in the k-mer case as well, with the only difference being that we need to account for directionalities (5'-3' vs. 3'-5') of the oligonucleotides. Hence, the rate constants of the substitutions that are reverse complementary to each other will be the same. We can see this on the example of ApT→ApT substitution.

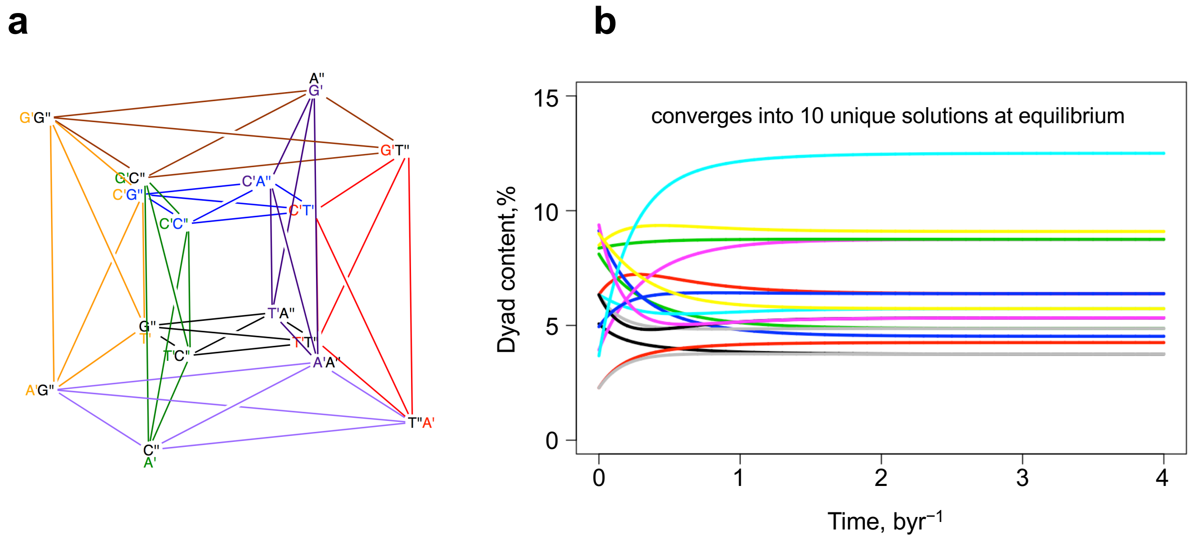

The NSB principle extended into dyads. *The schematic representation of the cross-mutation network among dyads (**a**) is constructed based on the tesseract (hypercube). There, the primed superscripts denote the position in the dyad, from 5' to 3' direction (A'G" is the same as ApG). Each line should be interpreted as a set of two counter-directed arrows. The models, along with the reduction of the number of independent rate constants are shown below. **b** shows an example of the time evolution of dyad contents, modelled without and with context dependence for mutation rates, starting from arbitrary sets of initial dyad contents and rate constants. The colouring scheme is also random. The convergence to unique 10 dyad content values can be noted in **b**.*

Taking into account that even the oligomeric mutations are prevalently driven by point mutations, [4] we can construct the network of cross-conversions between dyads on the basis of a tesseract (above Figure a), where the connections exist only between vertices that already have one common base. The superscript signs ' and " mark the first and the second bases, respectively, along the 5'-3' direction. Each line in the scheme should be interpreted as a set of two arrows in opposite directions. For this network, we can now construct the system of state equations by accounting for all the rate constant equalities, as described above for the dimeric case. The corresponding kinetic model is comprised of 16 equations for 16 unique dyads. In *Mathematica*, the system can be specified as:

```
DSolve[{CAA'[t] == kCA2AA∗CCA[t] − kAA2CA∗CAA[t] + kTA2AA∗CTA[t] − kAA2TA∗CAA[t] + kGA2AA∗CGA[t] −
    kAA2GA∗CAA[t] + kAC2AA∗CAC[t] − kAA2AC∗CAA[t] + kAT2AA∗CAT[t] − kAA2AT∗CAA[t] + kAG2AA∗CAG[t] −
    kAA2AG∗CAA[t],
CAC'[t] == kCC2AC∗CCC[t] − kAC2CC∗CAC[t] + kGA2GT∗CTC[t] − kAC2TC∗CAC[t] + kGC2AC∗CGC[t] − kAC2GC∗
    CAC[t] + kAA2AC∗CAA[t] − kAC2AA∗CAC[t] + kAT2AC∗CAT[t] − kAC2AT∗CAC[t] + kAG2AC∗CAG[t] − kAC2AG∗
    CAC[t],
CAG'[t] == kCG2AG∗CCG[t] − kAG2CG∗CAG[t] + kCA2CT∗CTG[t] − kAG2TG∗CAG[t] + kCC2CT∗CGG[t] − kAG2GG∗
    CAG[t] + kAA2AG∗CAA[t] − kAG2AA∗CAG[t] + kAC2AG∗CAC[t] − kAG2AC∗CAG[t] + kAT2AG∗CAT[t] − kAG2AT∗
```

```
    CAG[t],
CAT'[t] == kAG2AT*CCT[t] - kAT2AG*CAT[t] + kAA2AT*CTT[t] - kAT2AA*CAT[t] + kAC2AT*CGT[t] - kAT2AC*CAT
    [t] + kAA2AT*CAA[t] - kAT2AA*CAT[t] + kAC2AT*CAC[t] - kAT2AC*CAT[t] + kAG2AT*CAG[t] - kAT2AG*CAT[t
    ],
CCA'[t] == kAA2CA*CAA[t] - kCA2AA*CCA[t] + kTA2CA*CTA[t] - kCA2TA*CCA[t] + kGA2CA*CGA[t] - kCA2GA*
    CCA[t] + kCC2CA*CCC[t] - kCA2CC*CCA[t] + kAG2TG*CCT[t] - kCA2CT*CCA[t] + kCG2CA*CCG[t] - kCA2CG*
    CCA[t],
CCC'[t] == kAC2CC*CAC[t] - kCC2AC*CCC[t] + kGA2GG*CTC[t] - kCC2TC*CCC[t] + kGC2CC*CGC[t] - kCC2GC*
    CCC[t] + kCA2CC*CCA[t] - kCC2CA*CCC[t] + kAG2GG*CCT[t] - kCC2CT*CCC[t] + kCG2CC*CCG[t] - kCC2CG*
    CCC[t],
CCG'[t] == kAG2CG*CAG[t] - kCG2AG*CCG[t] + kCA2CG*CTG[t] - kCG2CA*CCG[t] + kCC2CG*CGG[t] - kCG2CC*
    CCG[t] + kCA2CG*CCA[t] - kCG2CA*CCG[t] + kCC2CG*CCC[t] - kCG2CC*CCG[t] + kAG2CG*CCT[t] - kCG2AG*
    CCG[t],
CCT'[t] == kAT2AG*CAT[t] - kAG2AT*CCT[t] + kAA2AG*CTT[t] - kAG2AA*CCT[t] + kAC2AG*CGT[t] - kAG2AC*
    CCT[t] + kCA2CT*CCA[t] - kAG2TG*CCT[t] + kCC2CT*CCC[t] - kAG2GG*CCT[t] + kCG2AG*CCG[t] - kAG2CG*
    CCT[t],
CGA'[t] == kAA2GA*CAA[t] - kGA2AA*CGA[t] + kCA2GA*CCA[t] - kGA2CA*CGA[t] + kTA2GA*CTA[t] - kGA2TA*
    CGA[t] + kGC2GA*CGC[t] - kGA2GC*CGA[t] + kAC2TC*CGT[t] - kGA2GT*CGA[t] + kCC2TC*CGG[t] - kGA2GG*
    CGA[t],
CGC'[t] == kAC2GC*CAC[t] - kGC2AC*CGC[t] + kCC2GC*CCC[t] - kGC2CC*CGC[t] + kGA2GC*CTC[t] - kGC2GA*
    CGC[t] + kGA2GC*CGA[t] - kGC2GA*CGC[t] + kAC2GC*CGT[t] - kGC2AC*CGC[t] + kCC2GC*CGG[t] - kGC2CC*
    CGC[t],
CGG'[t] == kAG2GG*CAG[t] - kCC2CT*CGG[t] + kCG2CC*CCG[t] - kCC2CG*CGG[t] + kCA2CC*CTG[t] - kCC2CA*
    CGG[t] + kGA2GG*CGA[t] - kCC2TC*CGG[t] + kGC2CC*CGC[t] - kCC2GC*CGG[t] + kAC2CC*CGT[t] - kCC2AC*
    CGG[t],
CGT'[t] == kAT2AC*CAT[t] - kAC2AT*CGT[t] + kAG2AC*CCT[t] - kAC2AG*CGT[t] + kAA2AC*CTT[t] - kAC2AA*
    CGT[t] + kGA2GT*CGA[t] - kAC2TC*CGT[t] + kGC2AC*CGC[t] - kAC2GC*CGT[t] + kCC2AC*CGG[t] - kAC2CC*
    CGT[t],
CTA'[t] == kAA2TA*CAA[t] - kTA2AA*CTA[t] + kCA2TA*CCA[t] - kTA2CA*CTA[t] + kGA2TA*CGA[t] - kTA2GA*
    CTA[t] + kGA2TA*CTC[t] - kTA2GA*CTA[t] + kAA2TA*CTT[t] - kTA2AA*CTA[t] + kCA2TA*CTG[t] - kTA2CA*
    CTA[t],
CTC'[t] == kAC2TC*CAC[t] - kGA2GT*CTC[t] + kCC2TC*CCC[t] - kGA2GG*CTC[t] + kGC2GA*CGC[t] - kGA2GC*
    CTC[t] + kTA2GA*CTA[t] - kGA2TA*CTC[t] + kAA2GA*CTT[t] - kGA2AA*CTC[t] + kCA2GA*CTG[t] - kGA2CA*
    CTC[t],
CTG'[t] == kAG2TG*CAG[t] - kCA2CT*CTG[t] + kCG2CA*CCG[t] - kCA2CG*CTG[t] + kCC2CA*CGG[t] - kCA2CC*
    CTG[t] + kTA2CA*CTA[t] - kCA2TA*CTG[t] + kGA2CA*CTC[t] - kCA2GA*CTG[t] + kAA2CA*CTT[t] - kCA2AA*
    CTG[t],
CTT'[t] == kAT2AA*CAT[t] - kAA2AT*CTT[t] + kAG2AA*CCT[t] - kAA2AG*CTT[t] + kAC2AA*CGT[t] - kAA2AC*
    CTT[t] + kTA2AA*CTA[t] - kAA2TA*CTT[t] + kGA2AA*CTC[t] - kAA2GA*CTT[t] + kCA2AA*CTG[t] - kAA2CA*
    CTT[t],
CAA + CAC + CAG + CAT + CCA + CCC + CCG + CCT + CGA + CGC + CGG + CGT + CTA + CTC + CTG + CTT
    == 1,
CAA[0] == CAA0, CAC[0] == CAC0, CAG[0] == CAG0, CAT[0] == CAT0,
CCA[0] == CCA0, CCC[0] == CCC0, CCG[0] == CCG0, CCT[0] == CCT0,
CGA[0] == CGA0, CGC[0] == CGC0, CGG[0] == CGG0, CGT[0] == CGT0,
CTA[0] == CTA0, CTC[0] == CTC0,CTG[0] == CTG0, CTT[0] == CTT0},
{CAA, CAC, CAG, CAT, CCA, CCC, CCG, CCT, CGA, CGC, CGG, CGT, CTA, CTC, CTG, CTT}, t] // FullSimplify
```

The equilibrium dimer fractions can therefore be computed *via*:

```
Solve[
kCA2AA*CCA - kAA2CA*CAA + kTA2AA*CTA - kAA2TA*CAA + kGA2AA*CGA - kAA2GA*CAA + kAC2AA*CAC -
    kAA2AC*CAA + kAT2AA*CAT - kAA2AT*CAA + kAG2AA*CAG - kAA2AG*CAA == 0 &&
kCC2AC*CCC - kAC2CC*CAC + kGA2GT*CTC - kAC2TC*CAC + kGC2AC*CGC - kAC2GC*CAC + kAA2AC*CAA -
    kAC2AA*CAC + kAT2AC*CAT - kAC2AT*CAC + kAG2AC*CAG - kAC2AG*CAC == 0 &&
kCG2AG*CCG - kAG2CG*CAG + kCA2CT*CTG - kAG2TG*CAG + kCC2CT*CGG - kAG2GG*CAG + kAA2AG*CAA -
    kAG2AA*CAG + kAC2AG*CAC - kAG2AC*CAG + kAT2AG*CAT - kAG2AT*CAG == 0 &&
kAG2AT*CCT - kAT2AG*CAT + kAA2AT*CTT - kAT2AA*CAT + kAC2AT*CGT - kAT2AC*CAT + kAA2AT*CAA -
    kAT2AA*CAT + kAC2AT*CAC - kAT2AC*CAT + kAG2AT*CAG - kAT2AG*CAT == 0 &&
kAA2CA*CAA - kCA2AA*CCA + kTA2CA*CTA - kCA2TA*CCA + kGA2CA*CGA - kCA2GA*CCA + kCC2CA*CCC -
    kCA2CC*CCA + kAG2TG*CCT - kCA2CT*CCA + kCG2CA*CCG - kCA2CG*CCA == 0 &&
kAC2CC*CAC - kCC2AC*CCC + kGA2GG*CTC - kCC2TC*CCC + kGC2CC*CGC - kCC2GC*CCC + kCA2CC*CCA -
    kCC2CA*CCC + kAG2GG*CCT - kCC2CT*CCC + kCG2CC*CCG - kCC2CG*CCC == 0 &&
kAG2CG*CAG - kCG2AG*CCG + kCA2CG*CTG - kCG2CA*CCG + kCC2CG*CGG - kCG2CC*CCG + kCA2CG*CCA -
    kCG2CA*CCG + kCC2CG*CCC - kCG2CC*CCG + kAG2CG*CCT - kCG2AG*CCG == 0 &&
kAT2AG*CAT - kAG2AT*CCT + kAA2AG*CTT - kAG2AA*CCT + kAC2AG*CGT - kAG2AC*CCT + kCA2CT*CCA -
```

```
      kAG2TG*CCT + kCC2CT*CCC − kAG2GG*CCT + kCG2AG*CCG − kAG2CG*CCT == 0 &&
   kAA2GA*CAA − kGA2AA*CGA + kCA2GA*CCA − kGA2CA*CGA + kTA2GA*CTA − kGA2TA*CGA + kGC2GA*CGC −
      kGA2GC*CGA + kAC2TC*CGT − kGA2GT*CGA + kCC2TC*CGG − kGA2GG*CGA == 0 &&
   kAC2GC*CAC − kGC2AC*CGC + kCC2GC*CCC − kGC2CC*CGC + kGA2GC*CTC − kGC2GA*CGC + kGA2GC*CGA −
      kGC2GA*CGC + kAC2GC*CGT − kGC2AC*CGC + kCC2GC*CGG − kGC2CC*CGC == 0 &&
   kAG2GG*CAG − kCC2CT*CGG + kCG2CC*CCG − kCC2CG*CGG + kCA2CC*CTG − kCC2CA*CGG + kGA2GG*CGA −
      kCC2TC*CGG + kGC2CC*CGC − kCC2GC*CGG + kAC2CC*CGT − kCC2AC*CGG == 0 &&
   kAT2AC*CAT − kAC2AT*CGT + kAG2AC*CCT − kAC2AG*CGT + kAA2AC*CTT − kAC2AA*CGT + kGA2GT*CGA −
      kAC2TC*CGT + kGC2AC*CGC − kAC2GC*CGT + kCC2AC*CGG − kAC2CC*CGT == 0 &&
   kAA2TA*CAA − kTA2AA*CTA + kCA2TA*CCA − kTA2CA*CTA + kGA2TA*CGA − kTA2GA*CTA + kGA2TA*CTC −
      kTA2GA*CTA + kAA2TA*CTT − kTA2AA*CTA + kCA2TA*CTG − kTA2CA*CTA == 0 &&
   kAC2TC*CAC − kGA2GT*CTC + kCC2TC*CCC − kGA2GG*CTC + kGC2GA*CGC − kGA2GC*CTC + kTA2GA*CTA −
      kGA2TA*CTC + kAA2GA*CTT − kGA2AA*CTC + kCA2GA*CTG − kGA2CA*CTC == 0 &&
   kAG2TG*CAG − kCA2CT*CTG + kCG2CA*CCG − kCA2CG*CTG + kCC2CA*CGG − kCA2CC*CTG + kTA2CA*CTA −
      kCA2TA*CTG + kGA2CA*CTC − kCA2GA*CTG + kAA2CA*CTT − kCA2AA*CTG == 0 &&
   kAT2AA*CAT − kAA2AT*CTT + kAG2AA*CCT − kAA2AG*CTT + kAC2AA*CGT − kAA2AC*CTT + kTA2AA*CTA −
      kAA2TA*CTT + kGA2AA*CTC − kAA2GA*CTT + kCA2AA*CTG − kAA2CA*CTT == 0 &&
   CAA + CAC + CAG + CAT + CCA + CCC + CCG + CCT + CGA + CGC + CGG + CGT + CTA + CTC + CTG + CTT
      == 1,
   {CAA, CAC, CAG, CAT, CCA, CCC, CCG, CCT, CGA, CGC, CGG, CGT,
  CTA, CTC, CTG, CTT}] // FullSimplify
```

However, the above system of 17 equations with its 48 unique coefficients (unique after accounting for the rate constant equivalencies caused by double-strandedness of DNA) is very heavy for *Mathematica*. Adding different conditions, such as the rate constants being greater than 0, setting the dimer fractions being always greater than 0, restricting the solution region to different boundaries and trying all the matrix methodologies discussed above, does not help in getting solutions with 24GB of RAM and 12 CPUs. By exploring the solutions for an arbitrary set of numeric inputs, one can, however, see the equivalencies in the pairs of evaluated C_{ij} dimer contents that are reverse complementary to each other. This is exactly what the extended version of the Chargaff's second parity rule states, which means that the oligo version of the observed species-invariant base-count equivalencies in genomes are also the result of the intrinsic rate constant constraints in the cross-mutation networks. Besides the numeric trials, the equivalency is clear from the comparison of the state equations for the reverse-complementary pairs. As an example, let us consider the state equations for the equilibrium fractions of ApA and its reverse complementary TpT dimers. After slight reorganisations, the equations for C_{AA} and C_{TT} correspondingly look like:

```
   {kCA2AA*CCA − kAA2CA*CAA + kTA2AA*CTA − kAA2TA*CAA + kGA2AA*CGA − kAA2GA*CAA + kAC2AA*CAC −
      kAA2AC*CAA + kAT2AA*CAT − kAA2AT*CAA + kAG2AA*CAG − kAA2AG*CAA == 0,

   kCA2AA*CTG − kAA2CA*CTT + kTA2AA*CTA − kAA2TA*CTT + kGA2AA*CTC −kAA2GA*CTT + kAC2AA*CGT −
      kAA2AC*CTT + kAT2AA*CAT − kAA2AT*CTT + kAG2AA*CCT − kAA2AG*CTT == 0}
```

Those two equations have exactly the same set of rate constants and the same overall values of the expressions. Hence, one of the solutions to this pair would be the set where $\{C_{CA} = C_{TG}, C_{AA} = C_{TT}, C_{GA} = C_{TC}, C_{AC} = C_{GT}, C_{AG} = C_{CT}\}$. Similar equalities can be inferred from the other 5 pairs of equations that have exactly the same set of rate constants (see below for the complete pairings).

```
   (*pair for CAA and CTT*)
   kCA2AA*CCA − kAA2CA*CAA + kTA2AA*CTA − kAA2TA*CAA + kGA2AA*CGA − kAA2GA*CAA + kAC2AA*CAC −
      kAA2AC*CAA + kAT2AA*CAT − kAA2AT*CAA + kAG2AA*CAG − kAA2AG*CAA == 0 &&
   kAT2AA*CAT − kAA2AT*CTT + kAG2AA*CCT − kAA2AG*CTT + kAC2AA*CGT − kAA2AC*CTT + kTA2AA*CTA −
      kAA2TA*CTT + kGA2AA*CTC − kAA2GA*CTT + kCA2AA*CTG − kAA2CA*CTT == 0

   (*pair for CAC and CGT*)
   kCC2AC*CCC − kAC2CC*CAC + kGA2GT*CTC − kAC2TC*CAC + kGC2AC*CGC − kAC2GC*CAC + kAA2AC*CAA −
      kAC2AA*CAC + kAT2AC*CAT − kAC2AT*CAC + kAG2AC*CAG − kAC2AG*CAC == 0 &&
   kAT2AC*CAT − kAC2AT*CGT + kAG2AC*CCT − kAC2AG*CGT + kAA2AC*CTT − kAC2AA*CGT + kGA2GT*CGA −
      kAC2TC*CGT + kGC2AC*CGC − kAC2GC*CGT + kCC2AC*CGG − kAC2CC*CGT == 0

   (*pair for CAG and CCT*)
```

```
kCG2AG*CCG - kAG2CG*CAG + kCA2CT*CTG - kAG2TG*CAG + kCC2CT*CGG - kAG2GG*CAG + kAA2AG*CAA -
      kAG2AA*CAG + kAC2AG*CAC - kAG2AC*CAG + kAT2AG*CAT - kAG2AT*CAG == 0 &&
kAT2AG*CAT - kAG2AT*CCT + kAA2AG*CTT - kAG2AA*CCT + kAC2AG*CGT - kAG2AC*CCT + kCA2CT*CCA -
      kAG2TG*CCT + kCC2CT*CCC - kAG2GG*CCT + kCG2AG*CCG - kAG2CG*CCT == 0

(*unpaired CAT*)
kAG2AT*CCT - kAT2AG*CAT + kAA2AT*CTT - kAT2AA*CAT + kAC2AT*CGT - kAT2AC*CAT + kAA2AT*CAA -
      kAT2AA*CAT + kAC2AT*CAC - kAT2AC*CAT + kAG2AT*CAG - kAT2AG*CAT == 0

(*pair for CCA and CTG*)
kAA2CA*CAA - kCA2AA*CCA + kTA2CA*CTA - kCA2TA*CCA + kGA2CA*CGA - kCA2GA*CCA + kCC2CA*CCC -
      kCA2CC*CCA + kAG2TG*CCT - kCA2CT*CCA + kCG2CA*CCG - kCA2CG*CCA == 0 &&
kAG2TG*CAG - kCA2CT*CTG + kCG2CA*CCG - kCA2CG*CTG + kCC2CA*CGG - kCA2CC*CTG + kTA2CA*CTA -
      kCA2TA*CTG + kGA2CA*CTC - kCA2GA*CTG + kAA2CA*CTT - kCA2AA*CTG == 0

(*pair for CCC and CGG*)
kAC2CC*CAC - kCC2AC*CCC + kGA2GG*CTC - kCC2TC*CCC + kGC2CC*CGC - kCC2GC*CCC + kCA2CC*CCA -
      kCC2CA*CCC + kAG2GG*CCT - kCC2CT*CCC + kCG2CC*CCG - kCC2CG*CCC == 0 &&
kAG2GG*CAG - kCC2CT*CGG + kCG2CC*CCG - kCC2CG*CGG + kCA2CC*CTG - kCC2CA*CGG + kGA2GG*CGA -
      kCC2TC*CGG + kGC2CC*CGC - kCC2GC*CGG + kAC2CC*CGT - kCC2AC*CGG == 0

(*unpaired CCG*)
kAG2CG*CAG - kCG2AG*CCG + kCA2CG*CTG - kCG2CA*CCG + kCC2CG*CGG - kCG2CC*CCG + kCA2CG*CCA -
      kCG2CA*CCG + kCC2CG*CCC - kCG2CC*CCG + kAG2CG*CCT - kCG2AG*CCG == 0

(*pair for CGA and CTC*)
kAA2GA*CAA - kGA2AA*CGA + kCA2GA*CCA - kGA2CA*CGA + kTA2GA*CTA - kGA2TA*CGA + kGC2GA*CGC -
      kGA2GC*CGA + kAC2TC*CGT - kGA2GT*CGA + kCC2TC*CGG - kGA2GG*CGA == 0 &&
kAC2TC*CAC - kGA2GT*CTC + kCC2TC*CCC - kGA2GG*CTC + kGC2GA*CGC - kGA2GC*CTC + kTA2GA*CTA -
      kGA2TA*CTC + kAA2GA*CTT - kGA2AA*CTC + kCA2GA*CTG - kGA2CA*CTC == 0

(*unpaired CGC*)
kAC2GC*CAC - kGC2AC*CGC + kCC2GC*CCC - kGC2CC*CGC + kGA2GC*CTC - kGC2GA*CGC + kGA2GC*CGA -
      kGC2GA*CGC + kAC2GC*CGT - kGC2AC*CGC + kCC2GC*CGG - kGC2CC*CGC == 0

(*unpaired CTA*)
kAA2TA*CAA - kTA2AA*CTA + kCA2TA*CCA - kTA2CA*CTA + kGA2TA*CGA - kTA2GA*CTA + kGA2TA*CTC -
      kTA2GA*CTA + kAA2TA*CTT - kTA2AA*CTA + kCA2TA*CTG - kTA2CA*CTA == 0
```

In summary, we have extended the cross-mutation network to represent the di-nucleotide (dyad) fractions in genomes. The set of rate constants in this extension are now accounting for the neighbouring nucleotide effects. However, we demonstrated that the rate constant symmetries, analogous to the single-base case, apply to the oligomeric extension as well. We then wrote down the system of differential equations *via* the reduced 48 mutation rate constants. Thus, the corresponding kinetic model, which is comprised of 16 equations for 16 unique dyads, always equilibrates into 10 unique solutions (above Figure b), fully complying with the oligo-version of PR-2, where the counts of the reverse complementary oligomers are also equal to each other in a single strand.

If we assume no context dependence for the mutation rate constants, we can use the same i, j, k, l, m, n rate constants from the 1mer-NSB model in section to describe all the cross mutations in the hypercube-based cross-mutation model. Of course, this assumption is crude, especially for some dyads where the neighbouring effect can be substantial (for instance the bases in CpG dyads have substantially higher substitution rates). However, this relatively reduced dimeric model may still be useful to describe the overall dyad contents in genomes and to reveal the magnitude of neighbour effects on substitution rates. The system of dyad-state equations therefore reduces into the following:

```
DSolve[
  {CAA'[t] == (CAC[t] + CCA[t])*i + (CAT[t] + CTA[t])*l + (CAG[t] + CGA[t])*m - 2*CAA[t]*(j + l + n),
   CAC'[t] == CCC[t]*i + CAA[t]*j + CAG[t]*k + CTC[t]*l + CGC[t]*m + CAT[t]*n -
   CAC[t]*(i + j + k + l + m + n),
   CAG'[t] == CCG[t]*i + CAT[t]*j + CAC[t]*k + CTG[t]*l + CGG[t]*m + CAA[t]*n - CAG[t]*(i + j + k + l + m + n),
   CAT'[t] == (CAG[t] + CCT[t])*i + (CAA[t] + CTT[t])*l + (CAC[t] + CGT[t])*m - 2*CAT[t]*(j + l + n),
```

```
CCA'[t] == CCC[t]*i + CAA[t]*j + CGA[t]*k + CCT[t]*l + CCG[t]*m + CTA[t]*n −
CCA[t]*(i + j + k + l + m + n),
CCC'[t] == (CAC[t] + CCA[t])*j + (CCG[t] + CGC[t])*k + (CCT[t] + CTC[t])*n − 2*CCC[t]*(i + k + m),
CCG'[t] == (CAG[t] + CCT[t])*j + (CCC[t] + CGG[t])*k + (CCA[t] + CTG[t])*n − 2*CCG[t]*(i + k + m),
CCT'[t] == CCG[t]*i + CAT[t]*j + CGT[t]*k + CCA[t]*l + CCC[t]*m + CTT[t]*n −
CCT[t]*(i + j + k + l + m + n),
CGA'[t] == CGC[t]*i + CTA[t]*j + CCA[t]*k + CGT[t]*l + CGG[t]*m + CAA[t]*n −
CGA[t]*(i + j + k + l + m + n),
CGC'[t] == (CGA[t] + CTC[t])*j + (CCC[t] + CGG[t])*k + (CAC[t] + CGT[t])*n − 2*CGC[t]*(i + k + m),
CGG'[t] == (CGT[t] + CTG[t])*j + (CCG[t] + CGC[t])*k + (CAG[t] + CGA[t])*n − 2*CGG[t]*(i + k + m),
CGT'[t] == CGG[t]*i + CTT[t]*j + CCT[t]*k + CGA[t]*l + CGC[t]*m + CAT[t]*n −
CGT[t]*(i + j + k + l + m + n),
CTA'[t] == (CGA[t] + CTC[t])*i + (CAA[t] + CTT[t])*l + (CCA[t] + CTG[t])*m − 2*CTA[t]*(j + l + n),
CTC'[t] == CGC[t]*i + CTA[t]*j + CTG[t]*k + CAC[t]*l + CCC[t]*m + CTT[t]*n −
CTC[t]*(i + j + k + l + m + n),
CTG'[t] == CGG[t]*i + CTT[t]*j + CTC[t]*k + CAG[t]*l + CCG[t]*m + CTA[t]*n −
CTG[t]*(i + j + k + l + m + n),
CTT'[t] == (CGT[t] + CTG[t])*i + (CAT[t] + CTA[t])*l + (CCT[t] + CTC[t])*m − 2*CTT[t]*(j + l + n)},
{CAA, CAC, CAG, CAT, CCA, CCC, CCG, CCT, CGA, CGC, CGG, CGT, CTA,
CTC, CTG , CTT}, t] // FullSimplify
```

At equilibrium:

```
eq4 = {
  i*CCA − j*CAA + l*CTA − l*CAA + m*CGA − n*CAA + i*CAC − j*CAA + l*CAT − l*CAA + m*CAG − n*CAA == 0,
  i*CCC − j*CAC + l*CTC − l*CAC + m*CGC − n*CAC + j*CAA − i*CAC + n*CAT − m*CAC + k*CAG − k*CAC == 0,
  i*CCG − j*CAG + l*CTG − l*CAG + m*CGG − n*CAG + n*CAA − m*CAG + k*CAC − k*CAG + j*CAT − i*CAG == 0,
  i*CCT − j*CAT + l*CTT − l*CAT + m*CGT − n*CAT + l*CAA − l*CAT + m*CAC − n*CAT + i*CAG − j*CAT == 0,
  j*CAA − i*CCA + n*CTA − m*CCA + k*CGA − k*CCA + i*CCC − j*CCA + l*CCT − l*CCA + m*CCG − n*CCA == 0,
  j*CAC − i*CCC + n*CTC − m*CCC + k*CGC − k*CCC + j*CCA − i*CCC + n*CCT − m*CCC + k*CCG − k*CCC == 0,
  j*CAG − i*CCG + n*CTG − m*CCG + k*CGG − k*CCG + n*CCA − m*CCG + k*CCC − k*CCG + j*CCT − i*CCG ==
      0,
  j*CAT − i*CCT + n*CTT − m*CCT + k*CGT − k*CCT + l*CCA − l*CCT + m*CCC − n*CCT + i*CCG − j*CCT == 0,
  n*CAA − m*CGA + k*CCA − k*CGA + j*CTA − i*CGA + i*CGC − j*CGA + l*CGT − l*CGA + m*CGG − n*CGA == 0,
  n*CAC − m*CGC + k*CCC − k*CGC + j*CTC − i*CGC + j*CGA − i*CGC + n*CGT − m*CGC + k*CGG − k*CGC == 0,
  n*CAG − m*CGG + k*CCG − k*CGG + j*CTG − i*CGG + n*CGA − m*CGG + k*CGC − k*CGG + j*CGT − i*CGG ==
      0,
  n*CAT − m*CGT + k*CCT − k*CGT + j*CTT − i*CGT + l*CGA − l*CGT + m*CGC − n*CGT + i*CGG − j*CGT == 0,
  l*CAA − l*CTA + m*CCA − n*CTA + i*CGA − j*CTA + i*CTC − j*CTA + l*CTT − l*CTA + m*CTG − n*CTA == 0,
  l*CAC − l*CTC + m*CCC − n*CTC + i*CGC − j*CTC + j*CTA − i*CTC + n*CTT − m*CTC + k*CTG − k*CTC == 0,
  l*CAG − l*CTG + m*CCG − n*CTG + i*CGG − j*CTG + n*CTA − m*CTG + k*CTC − k*CTG + j*CTT − i*CTG == 0,
  l*CAT − l*CTT + m*CCT − n*CTT + i*CGT − j*CTT + l*CTA − l*CTT + m*CTC − n*CTT + i*CTG − j*CTT == 0,
  CAA + CAC + CAG + CAT + CCA + CCC + CCG + CCT + CGA + CGC + CGG + CGT + CTA + CTC + CTG + CTT
      == 1};

var4 = {CAA, CAC, CAG, CAT, CCA, CCC, CCG, CCT, CGA, CGC, CGG, CGT, CTA, CTC, CTG, CTT};
```

This system is still complex for *Mathematica*, but we can find exact symbolic solutions by carefully exploring the emergent groups in the equations. First, let us just assign an arbitrary set of numerical values to the $\{i, j, k, l, m, n\}$ rate constants.

```
{i, j, k, l, m, n} = {1.2, 2.3, 1.5, 4.1, 3.8, 5.3};
```

We can now find the numerical solutions:

```
NSolve[eq4, var4]


{{
CAA −> 0.0393676, CAC −> 0.0598388, CAG −> 0.0598388, CAT −> 0.0393676,
CCA −> 0.0598388, CCC −> 0.0909549, CCG −> 0.0909549, CCT −> 0.0598388,
CGA −> 0.0598388, CGC −> 0.0909549, CGG −> 0.0909549, CGT −> 0.0598388,
```

```
    CTA -> 0.0393676, CTC -> 0.0598388, CTG -> 0.0598388, CTT -> 0.0393676}}

    Clear[i, j, k, l, m, n]
```

The numerical solution to the system with the arbitrary assigned coefficients implies that:
$(C_{AA} = C_{TT}) = (C_{AT}) = (C_{TA}) = a = 0.0393676$ in an arbitrary case
$(C_{AC} = C_{GT}) = (C_{AG} = C_{CT}) = (C_{CA} = C_{TG}) = (C_{GA} = C_{TC}) = b = 0.0598388$ in an arbitrary case
$(C_{CC} = C_{GG}) = (C_{CG}) = (C_{GC}) = c = 0.0909549$ in an arbitrary case

where, the brackets denote the equalities expected from the oligo version of the second parity rule. Hence, the empirical rule is, again, emergent from the solution of the system. We just have some additional equalities that are present under the assumption of no context dependence for the mutation rate constants. Therefore, for such hypothetical genomes, Chargaff could observe even more equalities, and the dimeric composition of the whole genome could have been described by just 3 values, *a, b* and *c.* Let us go on and use the found groups (invariant to the *i, j, k, l, m, n* numeric values, as soon as those are greater than 0) to further trim the system and get the symbolic solutions for *a, b* and *c.* The trimming of the system can be done by taking only one equation from each of the found three groups. The solutions to this model will be valuable as we can further compare the outcomes for real genomes with the actual dimeric contents, in which case the distortions from the theoretical values will reveal the presence of strong neighbouring effects on the mutation rates. We shall solve the reduced system by first creating the sparse arrays and solving the system of linear equations in a matrix convention. The solutions will be for the remaining C_{AA}, C_{AC}, C_{CC} dimer fractions that correspond to *a, b* and *c* above, representing the full solution to the complete system specified above.

```
eq4trim =
  With[
  {CTT = CAA, CAT = CAA, CTA = CAA, CGT = CAC, CAG = CAC, CCT = CAC, CCA = CAC, CTG = CAC, CGA = CAC,
       CTC = CAC, CGG = CCC, CCG = CCC, CGC = CCC},
  {i*CCA - j*CAA + l*CTA - l*CAA + m*CGA - n*CAA + i*CAC - j*CAA + l*CAT - l*CAA + m*CAG - n*CAA == 0,
  i*CCC - j*CAC + l*CTC - l*CAC + m*CGC - n*CAC + j*CAA - i*CAC + n*CAT - m*CAC + k*CAG - k*CAC == 0,
  j*CAC - i*CCC + n*CTC - m*CCC + k*CGC - k*CCC + j*CCA - i*CCC + n*CCT - m*CCC + k*CCG - k*CCC == 0,
  CAA + CAC + CAG + CAT + CCA + CCC + CCG + CCT + CGA + CGC + CGG + CGT + CTA + CTC + CTG + CTT
       == 1}];

  var4trim = {CAA, CAC, CCC};

  mx4trim = CoefficientArrays[eq4trim, var4trim]
```

We can convert the above into a substitution matrix, *M*, as follows:

$$M = \begin{bmatrix} -2j-2n & 2i+2m & 0 \\ j+n & -i-j-m-n & i+m \\ 0 & 2j+2n & -2i-2m \\ 4 & 8 & 4 \end{bmatrix} = \begin{bmatrix} 0 \\ 0 \\ 0 \\ 1 \end{bmatrix} \tag{7}$$

We can solve this equation in *Mathematica* with:

```
    LinearSolve[ mx4trim [[2]], -mx4trim [[1]] ] // FullSimplify
```

and obtain the following solutions:

$$\begin{aligned} [C_{ApA} = C_{TpT}] = [C_{ApT}] = [C_{TpA}] &= \frac{(i+m)^2}{4(i+j+m+n)^2} \\ [C_{ApC} = C_{GpT}] = [C_{ApG} = C_{CpT}] = [C_{CpA} = C_{TpG}] = [C_{GpA} = C_{TpC}] &= \frac{(i+m)(j+n)}{4(i+j+m+n)^2} \\ [C_{CpC} = C_{GpG}] = [C_{CpG}] = [C_{GpC}] &= \frac{(j+n)^2}{4(i+j+m+n)^2} \end{aligned} \tag{8}$$

The unique $\{a,\ b,\ c\}$ solution is found and can be easily verified by cross checking against the numerical solutions for any arbitrary set of non-0 and positive $\{i,\ j,\ k,\ l,\ m,\ n\}$ rate constants.

```
{i, j, k, l, m, n} = {1.2, 2.3, 1.5, 4.1, 3.8, 5.3};
```

which returns the same values obtained numerically with **NSolve** above:
a = 0.0393676 in an arbitrary case
b = 0.0598388 in an arbitrary case
c = 0.0909549 in an arbitrary case

We have shown that, in this approximation, the model always converges into three unique dyad counts at equilibrium, the expressions of which reflect the connection between the genomic dyad content and the underlying individual rate constants. As expected, the three solutions from 2mer-NSB without context dependence for the mutation rate constants are the cross multiplications of the two unique solutions obtained from 1mer-NSB.

Note S1.3. Application of the mutation rate constants under no-strand-bias in predicating singleton and dyad composition of chimpanzee genome

Jiang et al. used genome-wide dSNP data from the chimpanzee genome to infer different substitution fractions [5]. The ancestral states of the sites were deduced by comparing the dSNP containing sequences to the homologous ones in humans. The work also reported the normalised substitution fractions, where each base is found in equal 25% frequency. Those substitutions are a result of the mutations happening within approximately 5-7 million years (t) after the divergence from the human-chimp most recent common ancestor [6, 7]. Since t is relatively small, we can assume that the probability of the sites undergoing repeated mutations is negligible. Since the data are normalised into a uniform base content, n_i is always 0.25. Furthermore, both n_i and t will cancel out in the context of the equilibrium base content equations derived above. To this end, the normalised substitution fractions can be used in a manner similar to the rate constants, as their scaled versions. Below we shall take the reported values, calculate the overall equilibrium base contents (both monomeric and dimeric) in the chimpanzee genome, and compare those with the actual genomic data. The SNP-sequence-context-normalised substitution fractions, in %, for the chimpanzee genome (from the described publication) are:

$$\{fAG, fTC, fGA, fCT, fAC, fTG, fGT, fCA, fAT, fTA, fGC, fCG\} = \\ \{12.8, 12.7, 20.9, 21.1, 3.4, 3.4, 4.8, 4.9, 2.8, 2.8, 5.3, 5.2\}$$

where f_{ij} denotes the fraction of $i \to j$ base (single nucleotide) substitutions. We use these fractions as a replacement for the $\{i,\ j,\ k,\ l,\ m,\ n\}$ rate constants. First, we average the already negligible differences between the reported substitution fractions, which are supposed to be equal by the NSB rate constant equality described in section .

$$\begin{aligned} i &= (fCA + fGT)/2 \\ j &= (fAC + fTG)/2 \\ k &= (fCG + fGC)/2 \\ l &= (fAT + fTA)/2 \\ m &= (fCT + fGA)/2 \\ n &= (fAG + fTC)/2 \end{aligned} \tag{9}$$

Now we can calculate the equilibrium base contents of the chimpanzee genome using the cross-mutation

network solutions, where $\{C_A,\ C_G,\ C_T,\ C_C\}$ are the individual base contents, $\{a,\ b,\ c\}$ are the dimeric contents under the assumption of neighbour-invariant rate constants, where

$$\begin{aligned} (C_{\mathrm{AA}} = C_{\mathrm{TT}}) = (C_{\mathrm{AT}}) = (C_{\mathrm{TA}}) = a \\ (C_{\mathrm{AC}} = C_{\mathrm{GT}}) = (C_{\mathrm{AG}} = C_{\mathrm{CT}}) = (C_{\mathrm{CA}} = C_{\mathrm{TG}}) = (C_{\mathrm{GA}} = C_{\mathrm{TC}}) = b \\ (C_{\mathrm{CC}} = C_{\mathrm{GG}}) = (C_{\mathrm{CG}}) = (C_{\mathrm{GC}} = c \end{aligned} \tag{10}$$

which results in the following individual base contents

$$\begin{aligned} C_A &\to 0.307738 \\ C_G &\to 0.192262 \\ C_T &\to 0.307738 \\ C_C &\to 0.192262 \end{aligned}$$

and the following dimeric contents

$$\begin{aligned} a &\to 0.0947027 \\ b &\to 0.0591663 \\ c &\to 0.0369646 \end{aligned}$$

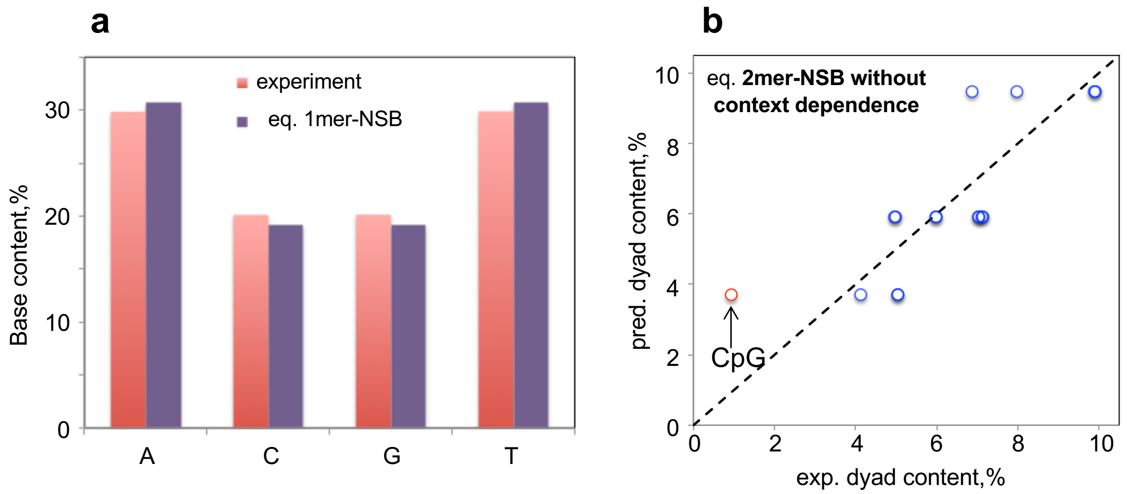

Application of the models on the chimpanzee genome. *Comparison of the equilibrium single base contents, predicted via 1mer-NSB, with the experimental one extracted from the reference genome,* ***a****. Correlation between the dyad contents is shown in* ***b****, where the prediction is done via 2mer-NSB without context dependence for the mutation rate constants.*

Unfortunately, we cannot apply the full hypercube model (without the neighbour invariance), which could have been applied numerically with NSolve in *Mathematica* if we would have the individual substitution fractions coming from 48 different dimer contexts. Comparing those with the experimental base and dyad contents of chimpanzee genome reveals a striking correlation. Since our solution is for the equilibrium genome, we can infer from the above Figure a, that the chimpanzee genome is slightly off equilibrium and will move towards it by having more G:C|C:G→A:T|T:A mutations, consistent with the prior view on the state of the chimpanzee genome [5]. As for comparing the predicted dyad contents, since we do not have the substitution fractions for the 48 independent dyad crossings to use those for the complete dyad inference (2mer-NSB), we have only used the solution to the hypercube model with the assumption of 2mer-NSB

without context dependence for the mutation rate constants by using the six rate constants inferred from the single-nucleotide-based substitution data. The agreement is still rather impressive (above Figure b), where, as expected, the neighbour effect distorts the CpG dyad frequency the most since the bases in CpG have much higher mutation rates owing to the involvement of epigenetic mechanisms. We expect these differences to vanish, as better-quality substitution data become available, accounting for the sequence context and enabling the usage of the full 2mer-NSB model for dyads.

Note S2. Machine learning model for classifying PR-2 compliance

Here, we developed a machine learning model to the non-symmetric, uniform distribution simulation outcome to fit a classifier for compliance and non-compliance with the PR-2 solutions. This would give an impression on the full potential of the mutation rate constants to proxy mirror the PR-2 compliance status without actually solving the ordinary differential equation systems. To do this, we used the tree-based extreme gradient boosting (XGBoost) machine learning model as our central framework for the model development [8, 9]. In gradient boosting, an ensemble of learners is developed with each iterative learner predicting the residual of the ensemble of prior learners. The combination of the decision trees (maximum interaction depth and minimum child weight) as the underlying learner with the gradient boosting process (number of boosted trees, learning rate, subsample percentage and gamma) allows flexible tunability and optimisation of the six hyperparameters; a combination that is commonly used in a wide range of machine learning competitions such as Kaggle (www.kaggle.com) and predictive modelling [8, 10]. Another important reason for selecting this machine learning strategy is that XGBoost allows the extraction of information regarding the importance of features, thereby giving us insight into which of the 12 mutation rate constants are most important to predict PR-2 compliance.

We employed the XGBoost machine learning model as the central framework for the model development in our study available in R through the caret library (https://cran.r-project.org/package=caret). For the machine learning model evaluation, we used the receiver operating characteristic (ROC) performance metric (using the MLeval library (https://cran.r-project.org/package=MLeval)) because, as we will describe in the following paragraph, our observations in the training set are balanced between each class. If it were skewed, other performance metrics may be more appropriate, such as the precision-recall curve [11–13]. We applied a k-fold cross validation (KCV) for evaluating the model (see Materials and Methods for details on hyperparameters). In a KCV procedure, the data set is randomly split into k smaller parts in order to reduce the risk of any over-fitting, and is a common practice for training machine learning models in the scientific literature [10, 14–17]. For instance, if k=5, the training data is split into 5 smaller sets, where, in each iteration, the hyperparameters of the model are trained using k-1 of the folds as the training data set. Next, the model is validated on the remaining part of the data set. Specifically, the remaining fold is used by the trained model as a test set to evaluate the performance metric, which, in our model, is the accuracy. This process is repeated k times, where, at the end of this process, the performance measure is reported as the average of the values in each k-fold. Given the size of the training data and common range of values for the k in KCV in the scientific literature, our model was trained with k=6 repeated once.

Using the tolerance values from eukaryotic organisms on the 25 million systems generated from the non-symmetric, uniform distribution simulation, we naturally end up with a disproportionate amount of non-compliant cases over the compliant cases. For the purpose of the machine learning process, we, therefore, used an equal number of compliant and non-compliant cases for the training set, starting from all 12 mutation rate constants as features. We also performed a principal component analysis on these 12 mutation rate constants for the equilibrated cases without imposing the PR-2 tolerance and found that there was no outstanding principal component (data not shown). The selection of non-compliant cases is done via random sampling ($seed = 2022$). We normalised the selected data set ($seed = 1234$) to randomly sample the seeds for each of the six cross-validation processes, repeated once. In the model training process, we tuned the learning parameters using a grid search approach. The performance metric selected for the development of the machine learning model was the receiver operator characteristic (ROC) curve, which shows the True Positive Rate (TPR) vs.

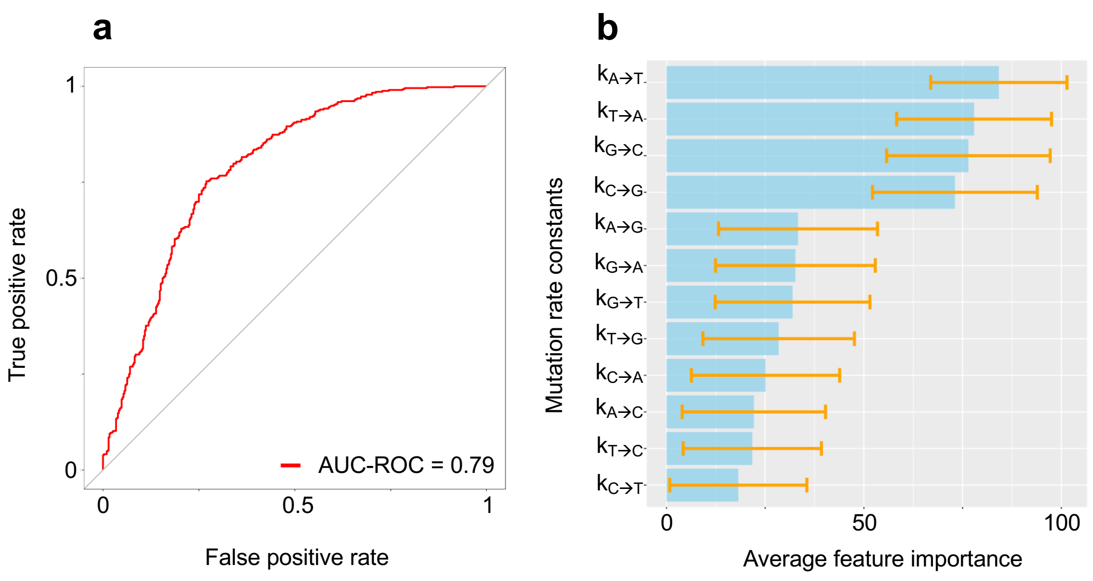

The performance of the final machine learning model strategy. *(**a**) Area under the receiver operating characteristic (AUC-ROC) curve showing the performance of the classifier for compliance and non-compliance with the PR-2 solutions. (**b**) The 12 mutation rate constant-based features ranked by relative average importance for the achieved prediction quality. The selection of non-compliant PR-2 cases was done via random sampling. As the model is trained on a random selection of non-compliant PR-2 cases, the feature importance of the 12 mutation rate constants would not be representative of the wider data set. Instead, we repeated the training process 1000 times using the optimised hyperparameters to obtain an average feature importance plot of the 12 mutation rate constants. The barplots represent the average feature importance and the orange bar represents the average ± 1 standard deviation.*

False Positive Rate (FPR) at varying classification thresholds. TPR is defined as the following:

$$TPR = \frac{TP}{TP + FN} \tag{11}$$

and FPR is defined as the following:

$$FPR = \frac{FP}{FP + TN} \tag{12}$$

To quantify the performance of the model across all possible classification thresholds, we compute the Area Under the ROC Curve (AUROC). The feature importance was calculated using the varImp function from the caret package. The optimised model obtained an AUROC value of 0.79 (above Figure a). Given the model is trained on a random selection of non-compliant PR-2 cases, the feature importance plot of the 12 mutation rate constants would not be representative of the wider data set. To circumvent this problem, we repeated the training process 1000 times using the optimised hyperparameters to obtain an average feature importance plot of the 12 mutation rate constants (below Table). For each random sampling of non-compliant PR-2 cases, we fixed the seed to 123. The diagonal transversion mutation rates ($k_{A\rightarrow T}$; $k_{T\rightarrow A}$; $k_{G\rightarrow C}$; $k_{C\rightarrow G}$) have a consistently higher feature importance compared to all other mutation rates (above Figure b).

Hyperparameters	Description	Tried values	Optimised model
Nrounds	Number of boosted trees to fit.	200, 500, 1000, 2000, 3000, 4000, 5000, 7000, 9000, 11000, 13000, 15000, 17000, 20000	17000
Max depth	Maximum depth of each tree.	5, 6, 8, 10	6
Gamma	Controls when to split a node.	0, 0.1, 0.2	0
Min child weight	Minimum sum of instance weight needed in a child.	1, 5, 10	1
ETA	Controls the contribution of each tree on the outcome and how quickly the algorithm learns.	0.005, 0.01, 0.02, 0.1	0.01
Subsample	Fraction of observations to be randomly selected for each tree.	0.3, 0.4, 0.6, 0.8	0.3
Colsample bytree	Subsample ratio of columns when constructing each tree.	1	1

Key hyperparameters tuned for the eXtreme Gradient Boosting (XGBoost) classification model.

Supplementary Figures

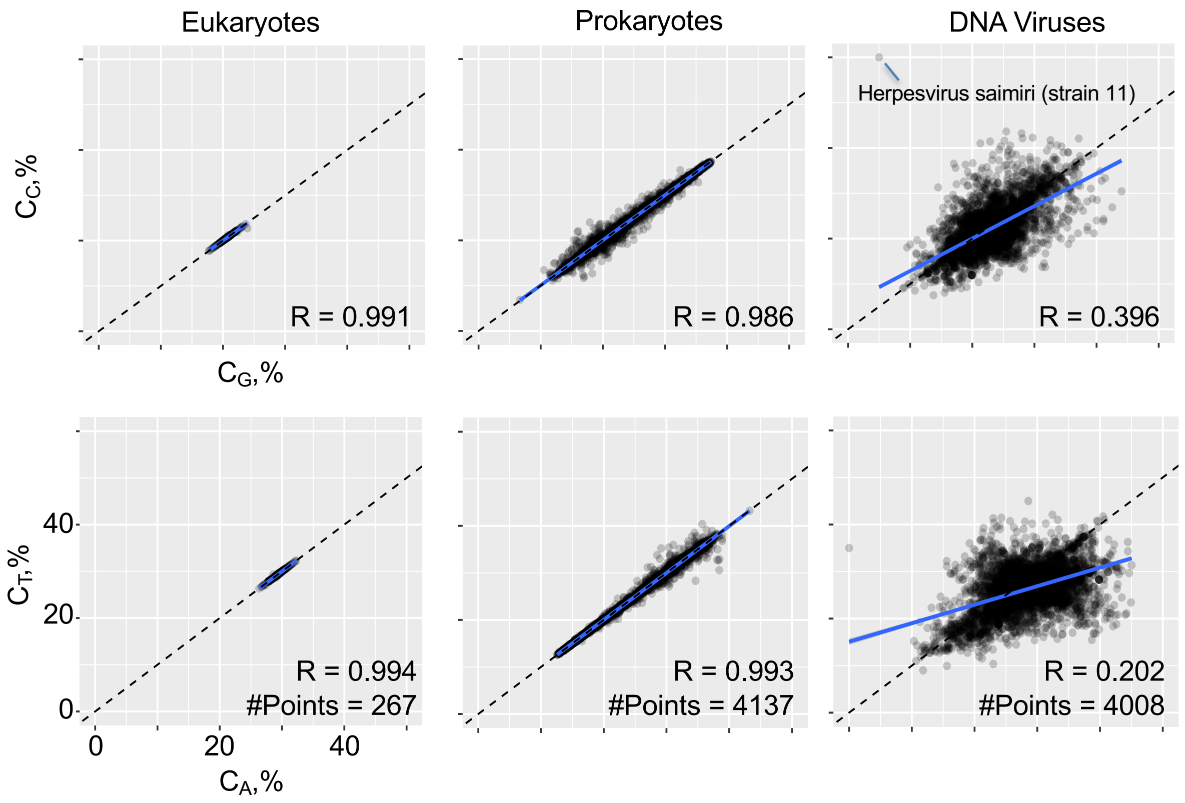

Figure S1. ***The percentage of guanine (G) vs. cytosine (C) and adenine (A) vs. thymine (T) computed for species in the eukaryotes, prokaryotes and DNA virus kingdoms****. Each of the base contents are computed on the reference strand for a given dsDNA genome. (**top row**) Percentage of the C_G vs. C_C contents represented as scatterplots. Eukaryotes have the highest Pearson correlation coefficient value (R = 0.991), prokaryotes similarly high (R = 0.986) while DNA viruses exhibit the lowest correlation coefficient value (R = 0.396). Following the filtering process for the species in each kingdom (see* ***Materials and Methods****), the genome of the herpesvirus saimiri (strain 11) has the most extreme base contents compared to the other species in the DNA virus kingdom. (**bottom row**) Similar graphs were generated for the percentage of the C_A vs. C_T contents. Eukaryotes have the highest Pearson correlation coefficient value (R = 0.994), prokaryotes similarly high (R = 0.993) while DNA viruses exhibit the lowest correlation coefficient value (R = 0.202).*

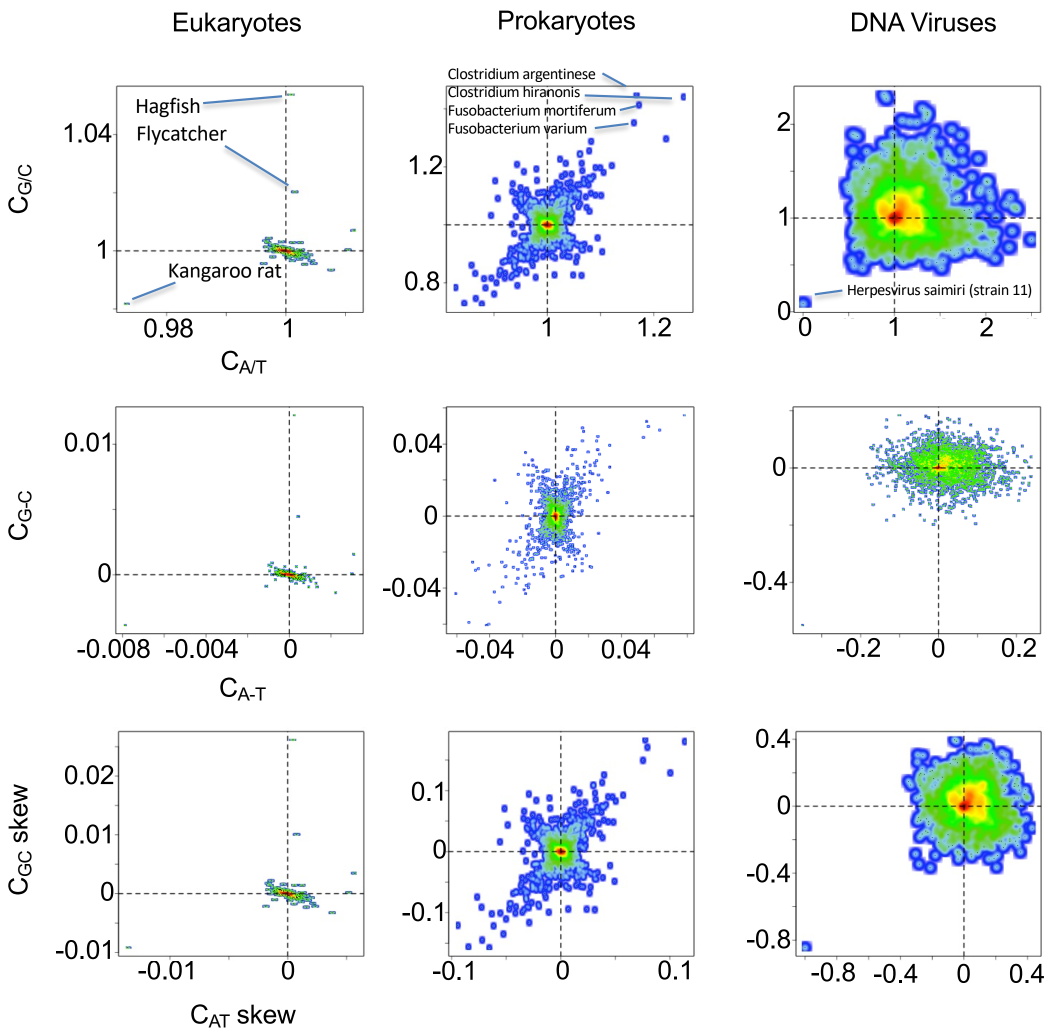

Figure S2. ***Nucleotide compositions for species in the eukaryotes, prokaryotes and DNA virus kingdoms***. *Each of the base contents are computed on the reference strand for a given dsDNA genome and represented as a 2-dimensional kernel density estimate scatterplot where the dotted vertical and horizontal lines indicate perfect parity. (**top row**) The base content ratio of $C_{G/C}$ vs. $C_{A/T}$ of eukaryotes have the tightest range with $C_{G/C}$ $\bar{x}$=1.000 s=3.890×10^{-3} and $C_{A/T}$ $\bar{x}$=1.000 s=2.343×10^{-3}. Prokaryotes are more dispersed compared to eukaryotes with $C_{G/C}$ $\bar{x}$=1.001 s=3.871×10^{-2} and $C_{A/T}$ $\bar{x}$=1.000 s=1.888×10^{-2}. DNA viruses are the most dispersed with $\bar{x}$=1.067 s=0.212 and $C_{A/T}$ $\bar{x}$=1.101 s=0.237. Following the filtering process for the species in each kingdom (see **Materials and Methods**), we highlight some species that have the most extreme nucleotide compositions for each of the three kingdoms. (**middle row**) The base content difference of $C_{G\text{-}C}$ vs. $C_{A\text{-}T}$ of eukaryotes are the smallest with $C_{G\text{-}C}$ $\bar{x}$=3.950×10^{-5} s=8.689×10^{-4} and $C_{A/T}$ $\bar{x}$=6.537×10^{-6} s=6.812×10^{-4}. Prokaryotes are more dispersed with $C_{G\text{-}C}$ $\bar{x}$=4.474×10^{-5} s=7.611×10^{-3} and $C_{A\text{-}T}$ $\bar{x}$=-1.758×10^{-5} s=5.646×10^{-3}. DNA Viruses are the most dispersed with $C_{G\text{-}C}$ $\bar{x}$=8.488×10^{-3} s=4.451×10^{-2} and $C_{A\text{-}T}$ $\bar{x}$=2.083×10^{-2} s=5.720×10^{-2}. (**bottom row**) The base content G+C content skew vs. C_{AT} skew of eukaryotes have the smallest G+C content skew $\bar{x}$=7.780×10^{-5} s=8.589×10^{-4} and C_{AT} skew $\bar{x}$=6.537×10^{-6} s=6.817×10^{-4}. Prokaryotes have a G+C content skew $\bar{x}$=1.413×10^{-4} s=1.907×10^{-2} and C_{AT} skew $\bar{x}$=-1.386×10^{-5} s=9.400×10^{-3}. If the data set of the prokaryotic organisms is split by the average G+C content of 51.16% and plot the G+C content vs. C_{AT} skew separately for the G+C content above and below the average, we observe the present diagonal pattern (bottom left to top right) for below average G+C content, while a diagonal pattern (top left to bottom right) for above average G+C content. DNA viruses have a G+C content skew $\bar{x}$=2.262×10^{-2} s=9.667×10^{-2} and C_{AT} skew $\bar{x}$=3.668×10^{-2} s=0.104.*

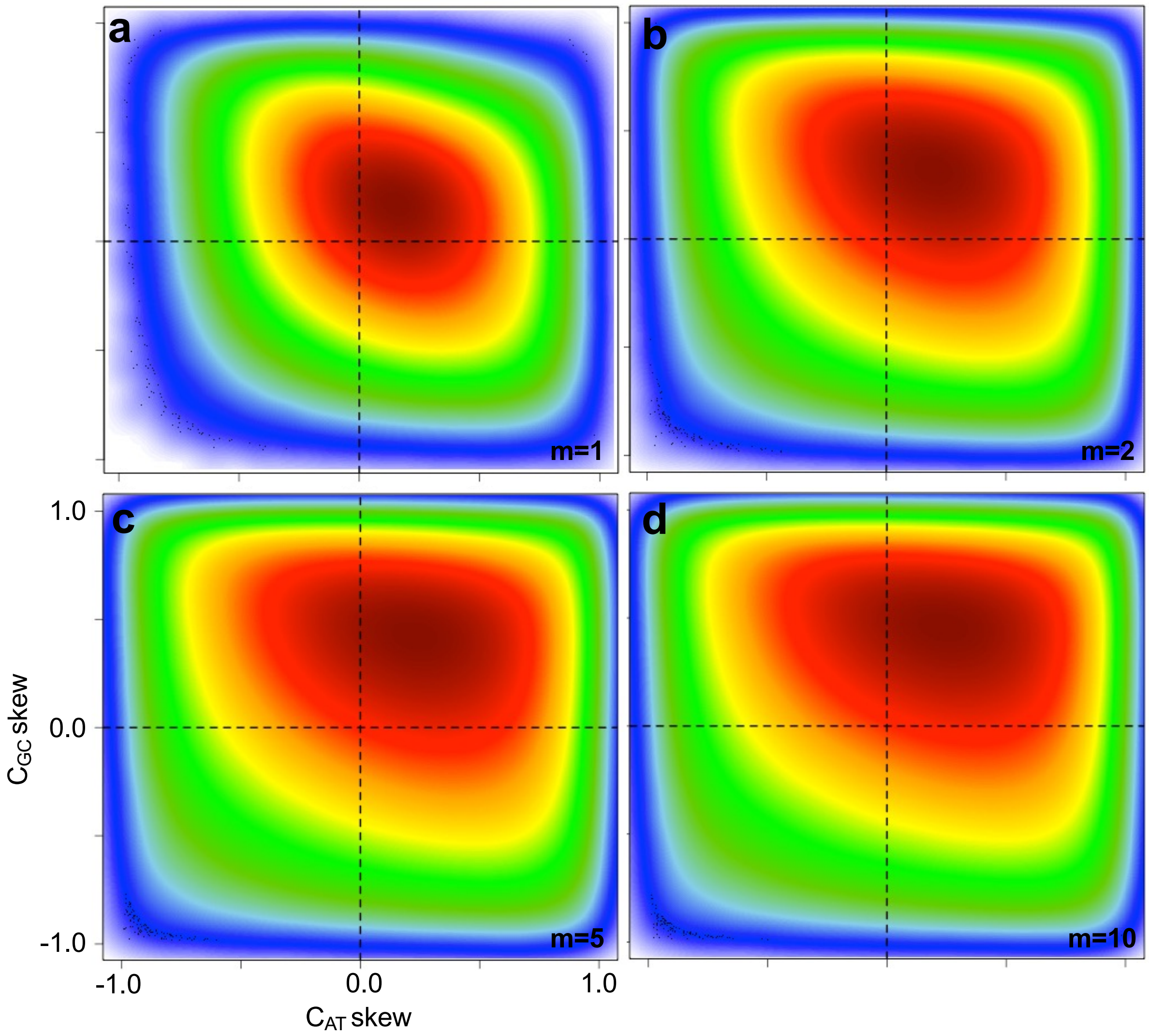

Figure S3. ***Numerical analysis of all-independent mutation rate constants assumed to have a normal distribution.*** *The systems of equations were solved starting from 25% initial contents for all four bases and rate constants randomly and independently drawn from a truncated normal distribution obtained from the Trek methodology [14] in byr-1 range. 25,000,000 such systems were calculated to produce genomic base contents at the final 4.28 byr time point. The 2-dimensional kernel density estimate scatterplots in a-d present the distribution of G+C content and C_{AT} content skews from the outcome of the simulation (colours vary with decreasing occurrence frequency from red to blue). The simulations were run four times separately with standard deviation values scaled by an additional multiplier $m \in \{1,2,5,10\}$ in plots a-d, respectively.*

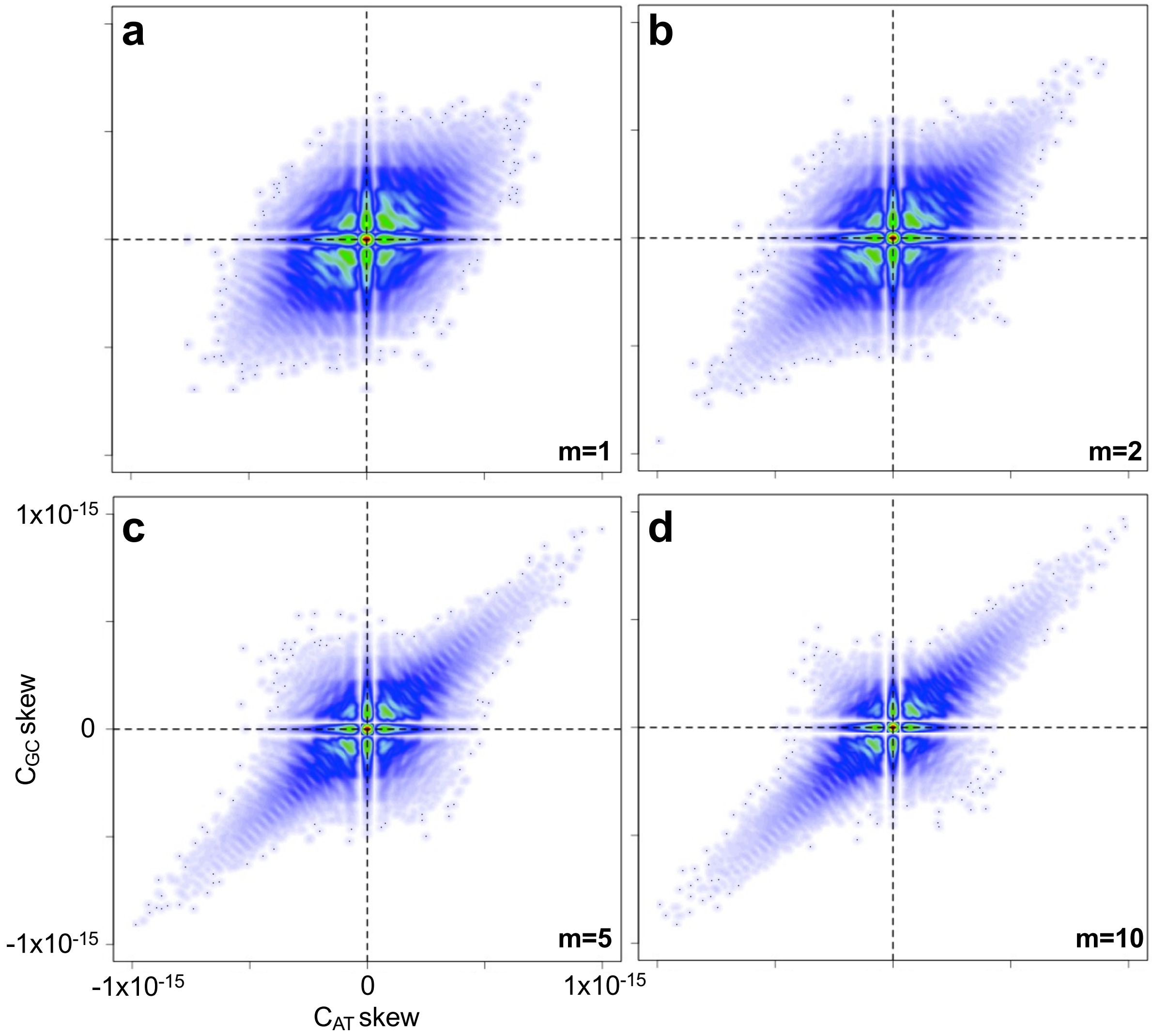

Figure S4. ***Numerical analysis of the NSB model symmetry-constrained mutation rate constants assumed to have a truncated normal distribution.*** *The systems of equations were solved starting from 25% initial contents for all four bases and symmetry-constrained rate constants randomly drawn from a truncated normal distribution obtained from the Trek methodology [14] in byr^{-1} range. 25,000,000 such systems were calculated to produce genomic base contents at the final 4.28 byr time point. The 2-dimensional kernel density estimate scatterplots in a-d present the distribution of G+C content and C_{AT} content skews from the outcome of the simulation (colours vary with decreasing occurrence frequency from red to blue). The simulations were run four times separately with standard deviation values additionally scaled by $m \in \{1,2,5,10\}$ multipliers in plots a-d, respectively.*

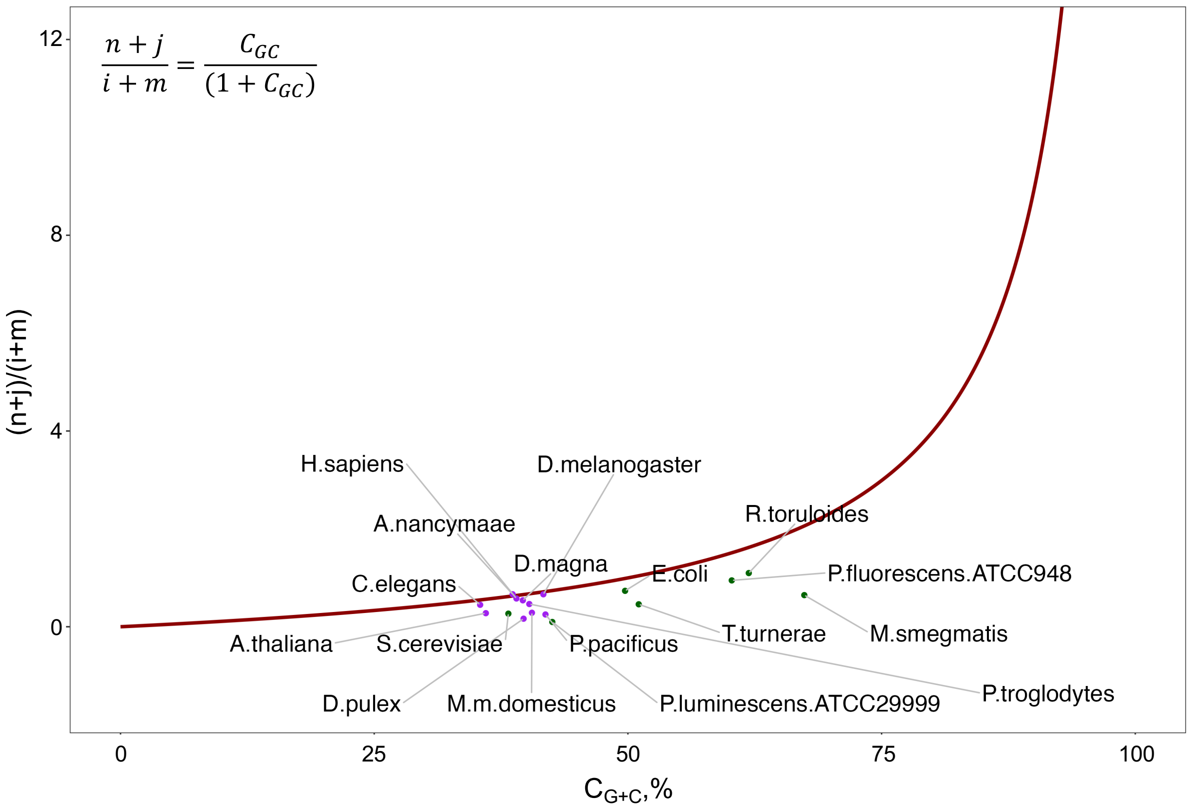

Figure S5. ***The base content solutions at equilibrium reveal the dependencies between the genomic G+C content content and the mutation rate constants (red line).*** *The strand symmetric mutation rate constants were obtained from 17 species across the eukaryotic and prokaryotic kingdoms [18–28] and overlaid as a scatterplot with colourings based on their associated eukaryotic (purple) or prokaryotic (dark green) kingdoms.*

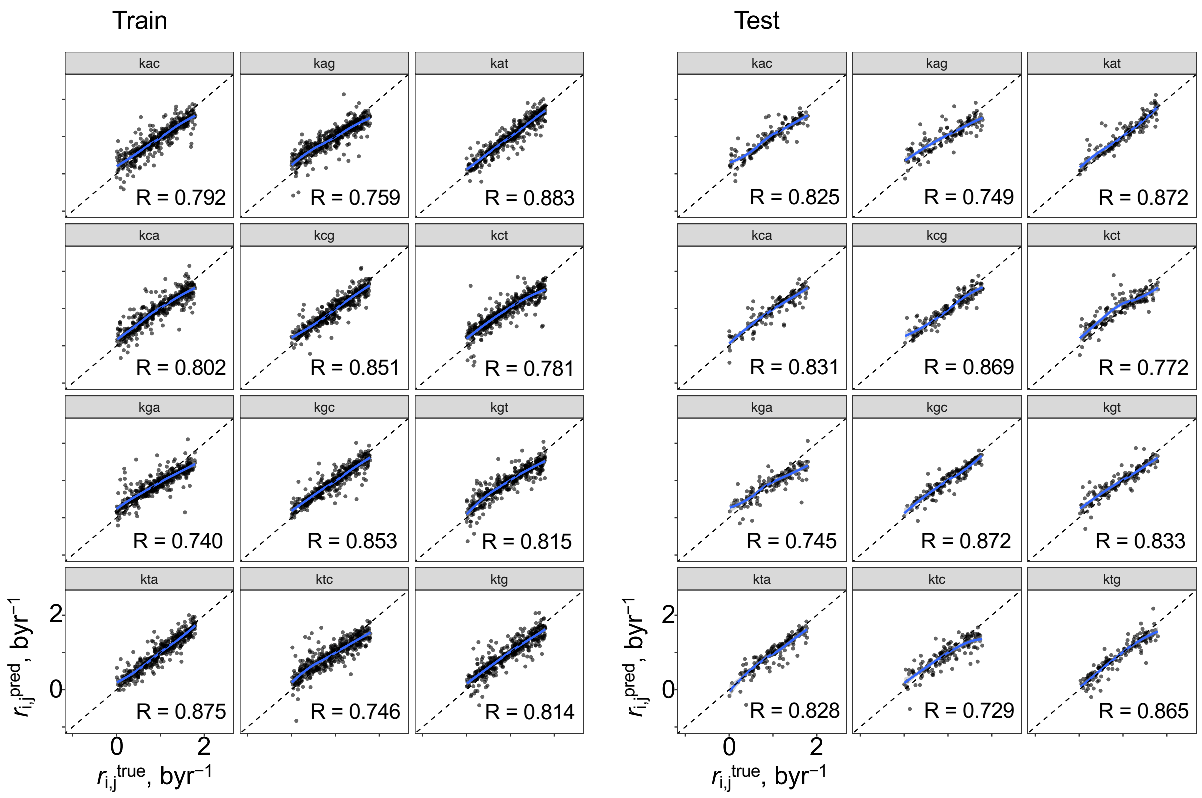

Figure S6. ***Evaluations of the analytical relations of the 12 independent mutation rate constants compliant with PR-2.*** *(**left**) Equations generated from Eureqa (now part of DataRobot) [29, 30] used to predict the mutation rate constant and comparing it against the mutation rate constant that arrived to the PR-2 compliant solution in the simulation. Pearson correlation coefficients are indicated on each graph where the equations for the* $k_{A\to T}$ *has the highest value (R = 0.883) while* $k_{G\to A}$ *has the lowest (R = 0.740). (**right**) Similar graphs were obtained for the test set (see main paper), where the equations for the* $k_{G\to C}$ *has the highest value (R = 0.872) (R = 0.872 for* $k_{A\to T}$*) while* $k_{T\to C}$ *has the lowest (R = 0.729). The diagonal dotted line represents perfect correlation with a slope of 1.*

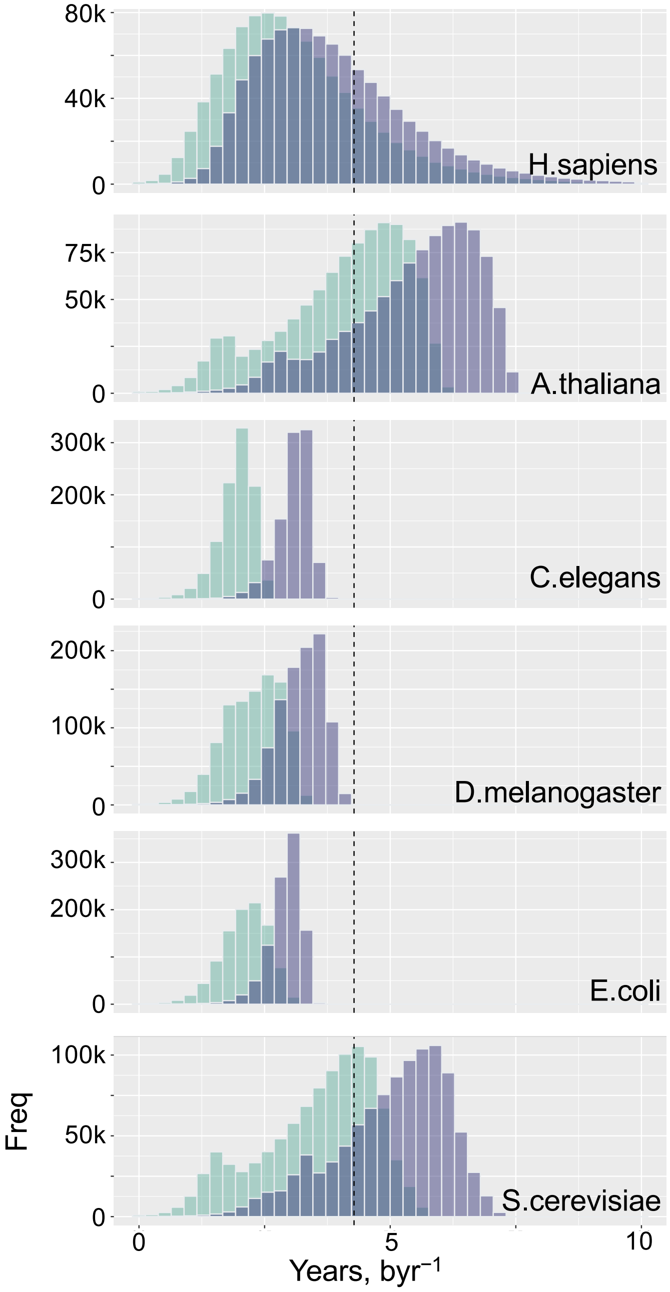

Figure S7. ***Distribution of time to reach PR-2 compliance and genome equilibrium.*** *10 million systems with the simulation model were generated where the initial base content was randomly sampled from the maximum allowed range in eukaryotic and prokaryotic organisms for the first two randomly sampled bases, with the remaining two bases being sampled from 1-remainder for the four bases to always sum to 1 for a complete genome. The strand symmetric-based mutation rate constants were randomly drawn from a truncated normal distribution, the mutation rate constants of which were obtained from work done by Michael Lynch [28]. This process was performed for each of the six species independently. The green histograms represent the distribution of time to reach PR-2 compliance and the purple histograms represent the distribution of time to reach genome equilibrium (see* ***Materials and Methods*** *for details). The vertical dotted line intercepts the x-axis at 4.28 byr, the maximum current estimate of age of life on Earth [31].*

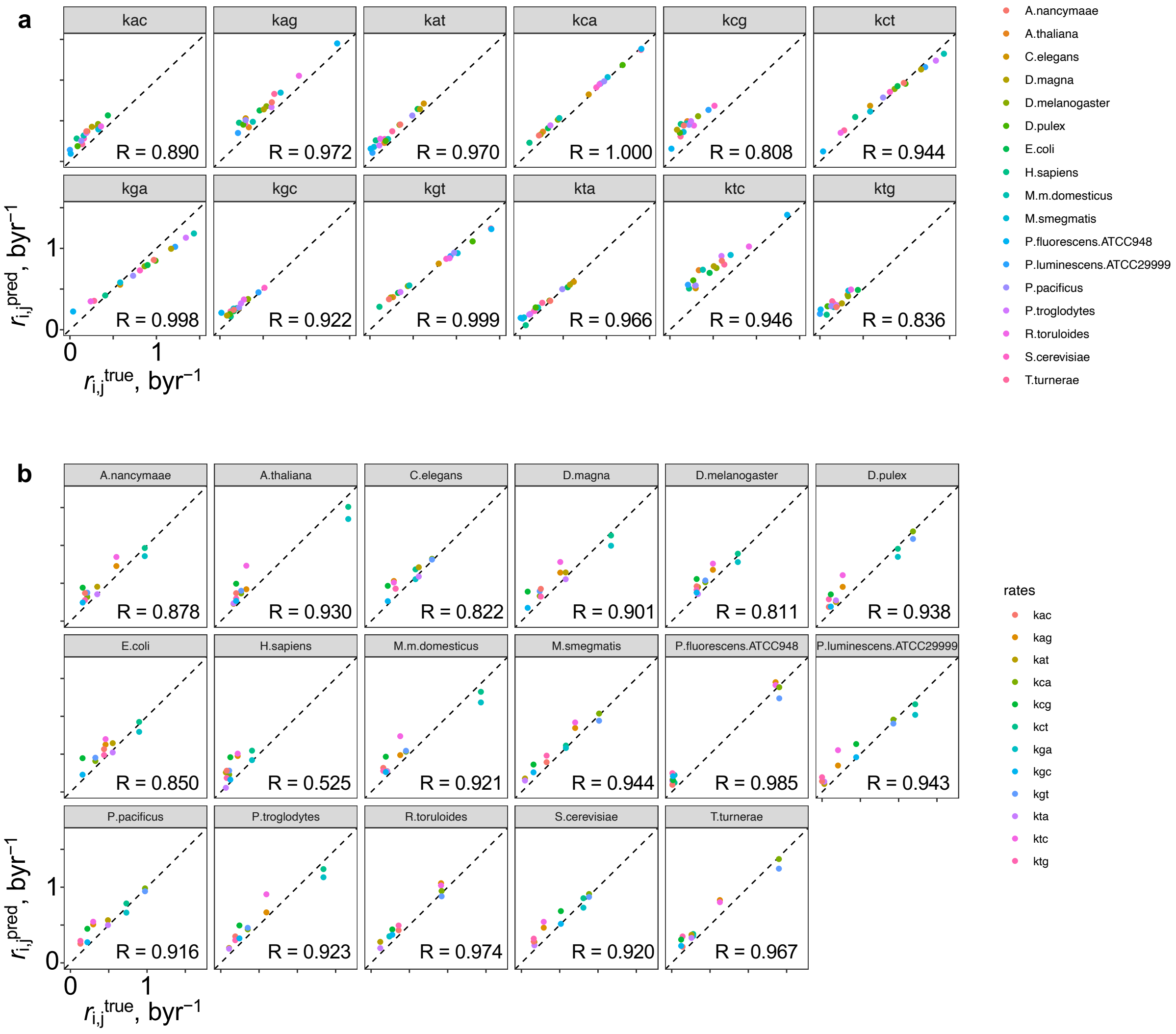

Figure S8. ***Examination of the closeness of current PR-2 compliant lifeforms to the fully equilibrated solution.(a)*** *The strand symmetric mutation rate constants were obtained from 17 species across the eukaryotic and prokaryotic kingdoms [18–28], aligned to the Trek-scaling [14] and substituted the parameters of the 12 sets of mutation rate constant equations. Each species is represents a unique colour. The Pearson correlation coefficient values are indicated on each graph where the* $k_{C\to A}$ *obtained the highest value (R = 1.000).* ***(b)*** *Similar graphs were obtained where each graph represents all the 12 mutation rate constants from* ***(a)*** *for each of the 17 species. The Homo sapiens species has the lowest Pearson correlation coefficient (R = 0.525). The diagonal dotted line represents perfect correlation with a slope of 1.*

References

[1] David N. Cooper et al. "The CpG dinucleotide and human genetic disease". In: *Human Genetics* 78.2 (Feb. 1988). Publisher: Springer, pp. 151–155. DOI: 10.1007/BF00278187.

[2] Adrian P. Bird. "DNA methylation and the frequency of CpG in animal DNA". In: *Nucleic Acids Research* 8.7 (Apr. 1980). Publisher: Oxford Academic, pp. 1499–1504. DOI: 10.1093/NAR/8.7.1499.

[3] Christine Coulondre et al. "Molecular basis of base substitution hotspots in Escherichia coli". In: *Nature* 274.5673 (1978). Publisher: Nature Publishing Group, pp. 775–780. DOI: 10.1038/274775a0.

[4] Michael W. Nachman et al. "Estimate of the mutation rate per nucleotide in Humans". In: *Genetics* 156.1 (Sept. 2000), pp. 297–304. DOI: 10.1093/genetics/156.1.297.

[5] Cizhong Jiang et al. "Directionality of point mutation and 5-methylcytosine deamination rates in the chimpanzee genome". In: *BMC Genomics* 7.1 (Dec. 2006). Publisher: BioMed Central, pp. 1–13. DOI: 10.1186/1471-2164-7-316.

[6] Zhao Z et al. "Worldwide DNA sequence variation in a 10-kilobase noncoding region on human chromosome 22". In: *Proceedings of the National Academy of Sciences of the United States of America* 97.21 (Oct. 2000). Publisher: Proc Natl Acad Sci U S A, pp. 11354–11358. DOI: 10.1073/PNAS.200348197.

[7] Feng-Chi Chen et al. "Genomic divergences between Humans and other Hominoids and the effective population size of the common ancestor of Humans and Chimpanzees". In: *American Journal of Human Genetics* 68.2 (2001). Publisher: Elsevier, pp. 444–444. DOI: 10.1086/318206.

[8] Jerome H. Friedman. "Greedy function approximation: A gradient boosting machine." In: *Institute of Mathematical Statistics* 29.5 (Oct. 2001). Publisher: Institute of Mathematical Statistics, pp. 1189–1232. DOI: 10.1214/AOS/1013203451.

[9] Alexey Natekin et al. "Gradient boosting machines, a tutorial". In: *Frontiers in Neurorobotics* 7.DEC (2013). Publisher: Frontiers Media SA. DOI: 10.3389/FNBOT.2013.00021.

[10] Baoshan Ma et al. "Diagnostic classification of cancers using extreme gradient boosting algorithm and multi-omics data". In: *Computers in Biology and Medicine* 121 (June 2020). Publisher: Pergamon, pp. 103761–103761. DOI: 10.1016/J.COMPBIOMED.2020.103761.

[11] Jesse Davis et al. "The relationship between precision-recall and ROC curves". In: *ACM International Conference Proceeding Series* 148 (2006), pp. 233–240. DOI: 10.1145/1143844.1143874.

[12] László A. Jeni et al. "Facing imbalanced data - recommendations for the use of performance metrics". In: *Proceedings - 2013 Humaine Association Conference on Affective Computing and Intelligent Interaction, ACII 2013* (2013), pp. 245–251. DOI: 10.1109/ACII.2013.47.

[13] Bowen Song et al. "ROC operating point selection for classification of imbalanced data with application to computer-aided polyp detection in CT colonography". In: *International journal of computer assisted radiology and surgery* 9.1 (Jan. 2014). Publisher: NIH Public Access, pp. 79–79. DOI: 10.1007/S11548-013-0913-8.

[14] Aleksandr B. Sahakyan et al. "Single genome retrieval of context-dependent variability in mutation rates for human germline". In: *BMC Genomics* 18.1 (Jan. 2017). Publisher: BioMed Central. DOI: 10.1186/S12864-016-3440-5.

[15] Liangliang Liu et al. "An interpretable boosting model to predict side effects of analgesics for osteoarthritis". In: *BMC Systems Biology* 12.6 (Nov. 2018). Publisher: BioMed Central, pp. 29–38. DOI: 10.1186/S12918-018-0624-4.

[16] Aleksandr B. Sahakyan et al. "Machine learning model for sequence-driven DNA G-quadruplex formation". In: *Scientific Reports* 7.1 (Nov. 2017). Publisher: Nature Publishing Group, pp. 1–11. DOI: 10.1038/s41598-017-14017-4.

[17] Bruce G. Marcot et al. "What is an optimal value of k in k-fold cross-validation in discrete Bayesian network analysis?" In: *Computational Statistics* 36.3 (June 2020). Publisher: Springer, pp. 2009–2031. DOI: 10.1007/S00180-020-00999-9.

[18] Jiao Pan et al. "The insect-killing bacterium Photorhabdus luminescens has the lowest mutation rate among bacteria". In: *Mar Life Sci Technol* 3.1 (Feb. 2021), pp. 20–27. DOI: 10.1007/s42995-020-00060-0.

[19] Beth L Dumont. "Significant strain variation in the mutation spectra of inbred laboratory Mice". In: *Molecular Biology and Evolution* 36.5 (May 2019), pp. 865–874. DOI: 10.1093/molbev/msz026.

[20] Eddie K H Ho et al. "High and highly variable spontaneous mutation rates in Daphnia". In: *Molecular Biology and Evolution* 37.11 (Nov. 2020), pp. 3258–3266. DOI: 10.1093/molbev/msaa142.

[21] Gregg W. C. Thomas et al. "Reproductive longevity predicts mutation rates in Primates". In: *Current Biology* 28.19 (Oct. 2018), 3193–3197.e5. DOI: 10.1016/j.cub.2018.08.050.

[22] Marcus V X Senra et al. "An unbiased genome-wide view of the mutation rate and spectrum of the Endosymbiotic Bacterium Teredinibacter turnerae". In: *Genome Biology and Evolution* 10.3 (Mar. 2018), pp. 723–730. DOI: 10.1093/gbe/evy027.

[23] Jullien M. Flynn et al. "Spontaneous mutation accumulation in Daphnia pulex in selection-free vs. competitive environments". In: *Molecular Biology and Evolution* 34.1 (Jan. 2017), pp. 160–173. DOI: 10.1093/molbev/msw234.

[24] Hongan Long et al. "Similar mutation rates but highly diverse mutation spectra in Ascomycete and Basidiomycete Yeasts". In: *Genome Biology and Evolution* 8.12 (Dec. 2016), pp. 3815–3821. DOI: 10.1093/gbe/evw286.

[25] Sibel Kucukyildirim et al. "The rate and spectrum of spontaneous mutations in Mycobacterium smegmatis, a Bacterium naturally devoid of the postreplicative mismatch repair pathway". In: *G3 Genes—Genomes—Genetics* 6.7 (July 2016), pp. 2157–2163. DOI: 10.1534/g3.116.030130.

[26] Hongan Long et al. "Mutation rate, spectrum, topology, and context-dependency in the DNA mismatch repair-deficient Pseudomonas fluorescens ATCC948". In: *Genome Biology and Evolution* 7.1 (Jan. 2015), pp. 262–271. DOI: 10.1093/gbe/evu284.

[27] Andreas M Weller et al. "Opposing forces of A/T-biased mutations and G/C-biased gene conversions shape the genome of the Nematode Pristionchus pacificus". In: *Genetics* 196.4 (Apr. 2014), pp. 1145–1152. DOI: 10.1534/genetics.113.159863.

[28] Michael Lynch. "Rate, molecular spectrum, and consequences of human mutation". In: *PNAS* 107.3 (Jan. 2010). Publisher: National Academy of Sciences Section: Biological Sciences, pp. 961–968. DOI: 10.1073/pnas.0912629107.

[29] Michael D Schmidt et al. "Coevolution of fitness predictors". In: *IEEE Transactions on Evolutionary Computation* 12.6 (2008). DOI: 10.1109/TEVC.2008.919006.

[30] Michael Schmidt et al. "Distilling free-form natural laws from experimental data". In: *Science* 324.5923 (Apr. 2009), pp. 81–85. DOI: 10.1126/SCIENCE.1165893.

[31] Matthew S. Dodd et al. "Evidence for early life in Earth's oldest hydrothermal vent precipitates". In: *Nature* 543.7643 (Mar. 2017), pp. 60–64. DOI: 10.1038/nature21377.
